# The Hippo signaling pathway as a therapeutic target in Alzheimer’s disease

**DOI:** 10.1186/s13024-025-00891-4

**Published:** 2025-09-26

**Authors:** Doris Chen, Stella Wigglesworth-Littlewood, Frank J. Gunn-Moore

**Affiliations:** School of Biology, University of St Andrews, St Andrews, KY16 9TF UK

**Keywords:** Hippo signaling, Alzheimer’s disease, Neuronal death

## Abstract

The Hippo signaling pathway is well-known for its regulation of organ size, cell proliferation, apoptosis, and cell migration and differentiation. Recent studies have demonstrated that Hippo signaling also plays important roles in the nervous system, being involved in neuroinflammation, neuronal differentiation, and neuronal death and degeneration. As such, dysregulation of Hippo signaling, particularly of its core kinases MST1/2 and LATS1/2, has begun to attract attention in the Alzheimer’s disease (AD) field. Here, we discuss the therapeutic potential of targeting the Hippo pathway in AD by providing an overview of Hippo signaling with regards to its function in the nervous system, evidence for its dysregulation in AD patients and models, and recent studies involving genetic or pharmacological modulation of this pathway in AD.

## Graphical Abstract

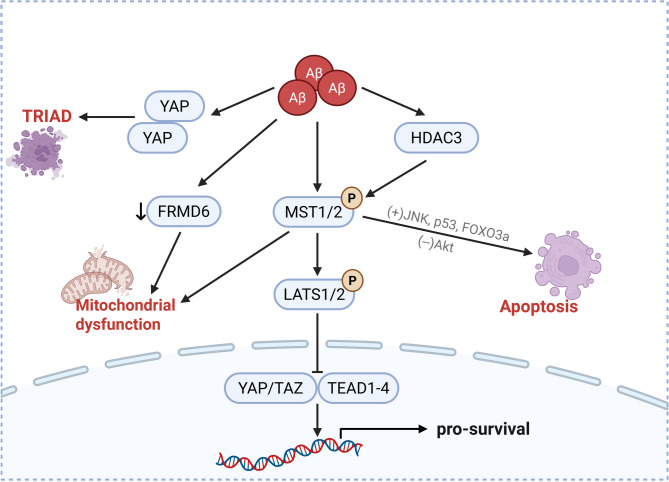

## Background

Alzheimer’s disease (AD) is a progressive neurodegenerative disorder characterized by cognitive decline, synaptic dysfunction, and neuronal loss [1, 2]. The complex pathogenesis of AD involves multiple cellular and molecular pathways, with the accumulation of amyloid-beta (Aβ) plaques and tau tangles playing central roles [3, 4]. Recent research has positioned the Hippo signaling pathway, traditionally recognized for its role in organ size regulation and tumor suppression [5, 6], as a key player in neurodegenerative diseases [7–13], including AD [14–25]. Dysregulation of Hippo signaling, particularly through hyperactivation of its core kinase MST1/2, has been implicated in promoting hallmarks of AD pathology such as neuroinflammation, oxidative stress, and neuronal death [26, 27]. Given the complex nature of AD pathogenesis which involves both biochemical and structural changes in the brain, a signaling cascade such as Hippo, which lacks a dedicated receptor and is therefore capable of integrating multiple upstream modalities [28], presents a promising therapeutic target. This review highlights recent research that addresses whether Hippo signaling is dysregulated in AD; how this dysregulation interacts with AD-relevant pathological changes; and emerging studies exploring the therapeutic potential of Hippo signaling inhibition, with a particular focus on MST1/2 inhibition, in AD.

## Main text

### The mammalian Hippo signaling pathway

**Fig. 1:**
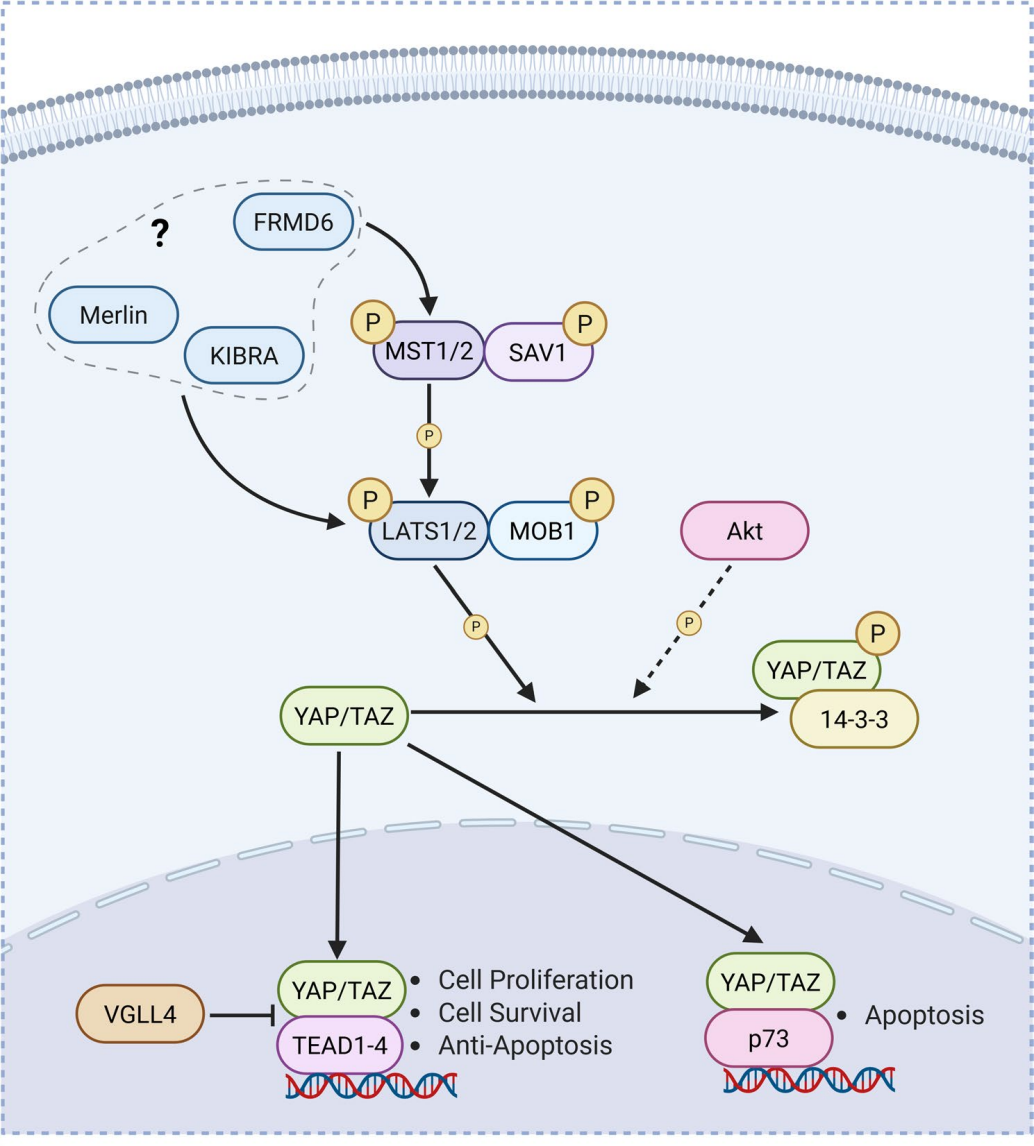
Overview of the mammalian Hippo signaling pathway. The Hippo pathway is a kinase cascade that responds to extracellular cues (such as cell density, stress, and mechanical factors) to influence transcription in order to control cell proliferation, differentiation, and apoptosis [29, 30]. During active Hippo signaling, MST1/2 kinases form a complex with SAV1 to phosphorylate and activate LATS1/2 kinases [35], which subsequently form a complex with MOB1 [269]. LATS1/2 kinases then phosphorylate transcription coactivators YAP and TAZ at S127 [31, 33] and S89 [57], respectively. Phosphorylation of YAP and TAZ leads to cytoplasmic sequestration through interaction with 14-3-3 proteins [31, 33, 57]. Unphosphorylated YAP/TAZ can translocate to the nucleus, where they regulate transcription through interaction with various transcription factors. YAP/TAZ interaction with TEAD1-4 leads to transcription of genes which promote cell proliferation and survival [270]. However, in response to cellular stress, including serum-starvation or DNA damage, YAP can instead bind p73, leading to pro-apoptotic gene expression [31, 45]. A number of proteins can regulate and influence the Hippo pathway. For example, FRMD6, Merlin, and KIBRA act as upstream regulators of the Hippo kinase cascade. Although their *Drosophila* homologs can form a complex to promote Hippo activation [271], their interaction is less well understood in mammals. In prostate cancer cells, KIBRA, Merlin, and FRMD6 form a complex to interact with and phosphorylate LATS1/2 [272]. However, they are also capable of acting independently of each other. FRMD6 promotes phosphorylation of MST1/2, LATS1, and YAP [86], potentially through direct binding to MST [87]. In contrast, both Merlin/NF2 [273] and KIBRA [39, 40] can promote LATS1/2 phosphorylation independently of MST1/2, but with different transcriptional outputs [40]. VGLL4 competitively binds with TEAD to inhibit YAP-TEAD signaling [29, 54, 55]. Akt can also phosphorylate YAP at S127, leading to nuclear exclusion and prevention of YAP-p73-mediated apoptosis [45], but this might be context-specific as this does not occur in some cell lines [33]

The Hippo signaling cascade (Fig. 1) integrates diverse upstream cues related to cell density, mechanical properties of the extracellular environment, actin remodeling, cell polarity, hormones and soluble factors, as well as cellular stress [29, 30]. These upstream signals converge upon the activation of the core Hippo kinases MST1/2 and LATS1/2 resulting in phosphorylation and cytoplasmic sequestration/degradation of the transcriptional co-activators YAP and TAZ in order to control organ size [5], regulate cell death [9, 31] and proliferation [6, 32, 33], and control cell migration and differentiation [28, 34].

**Fig. 2:**
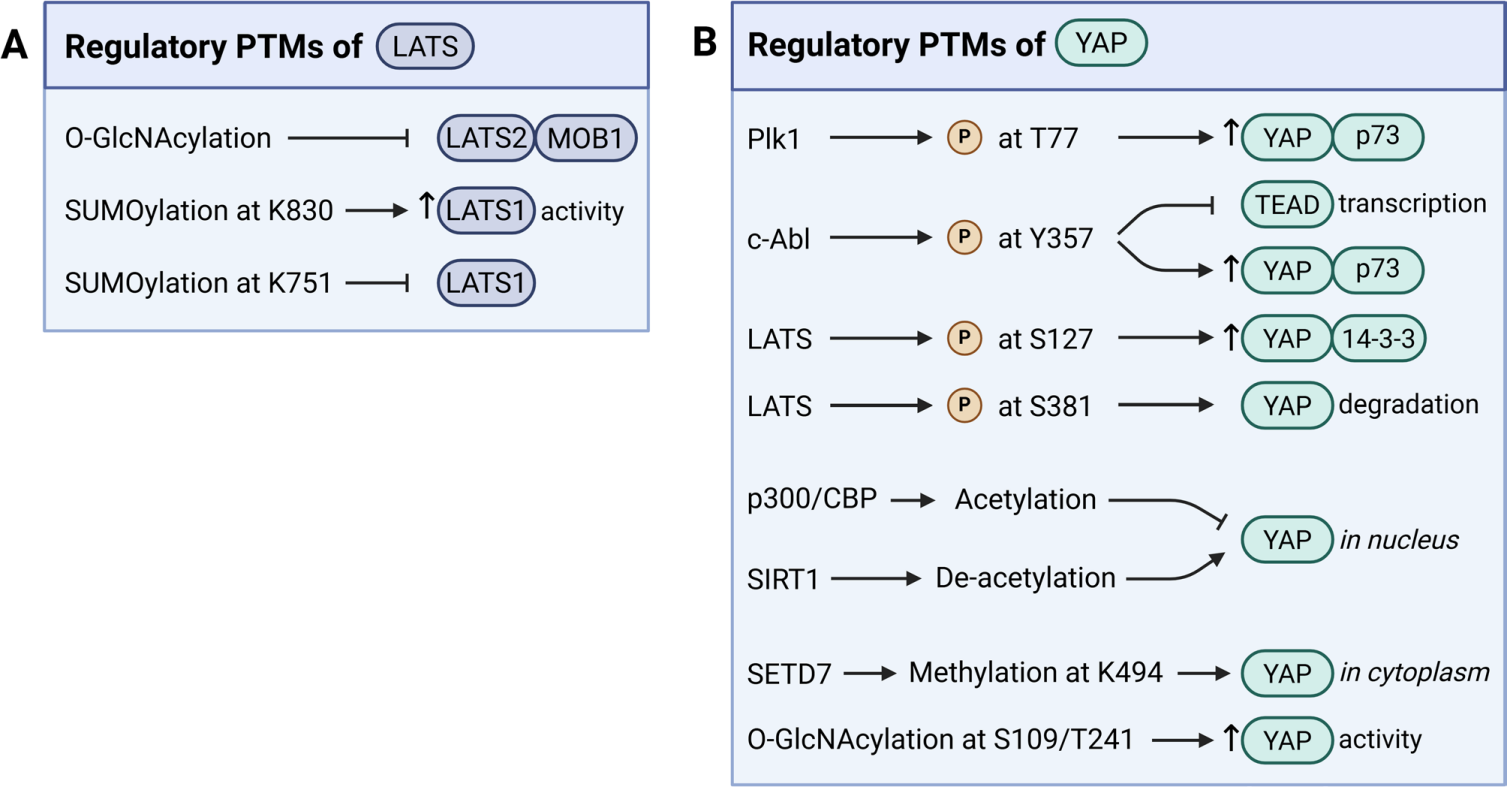
Regulatory mechanisms of Hippo signaling pathway components. Hippo pathway components can be regulated by a number of post-translational modifications (PTMs). **(A)** LATS can be regulated in an isoform-specific manner by non-phosphorylation PTMs. O-GlcNAcylation of LATS2 disrupts the LATS-MOB1 interaction, thus inhibiting LATS2 activity [41]. LATS1 can be SUMOylated: at K830, this leads to increased LATS activity [42], whereas SUMOylation at K751 has the opposite effect [43]. **(B)** PTMs can alter YAP subcellular localization, stability, and transcriptional activity. Phosphorylation of YAP at T77 by Plk1 leads to increased pro-apoptotic interaction between YAP and p73 [9]. c-Abl phosphorylation of YAP at Y357 promotes interaction with p73 [51] and inhibits TEAD transcription, and thus the expression of YAP-TEAD regulated genes [50]. Phosphorylation of YAP at S127 by LATS leads to increased interaction of YAP and 14-3-3, leading to YAP sequestration in the cytoplasm [31, 33, 45, 56]. LATS can also phosphorylate YAP at S381 to promote its proteasomal degradation [58]. YAP cellular localization is also affected by its acetylation, with acetylation by p300/CBP reducing nuclear YAP, and deacetylation by SIRT1 increasing nuclear YAP [62]. SETD7 methylates YAP at K494 leading to increased cytoplasmic YAP [63]. Additionally, YAP can under O-GlcNAcylation at S109 [64] and T241 [65], which increases its activity

The interaction between MST1/2 and LATS1/2 kinases is modulated by various adaptor and scaffolding proteins including SAV1 [35], MOB1A/B [36, 37], and KIBRA [38], although the detailed mechanisms underlying these interactions are not well understood. For example, while earlier studies using genetic depletion methods in HEK293T cells, as well as in tumorigenic MDA-MB-231 [39] and non-tumorigenic MCF10A [40] breast epithelial cells, demonstrated that KIBRA regulates Hippo signaling independently of MST1/2, a recent study using genetic deletion in HEK293a cells demonstrated that KIBRA could also modulate the strength of Hippo signaling by bringing LATS1/2 and the SAV1-MST1/2 complex into close proximity to facilitate LATS1/2 phosphorylation by MST1/2 [38]. In addition to phosphorylation, other regulatory post-translational modifications (PTMs) have been reported for LATS1/2 (Fig. 2A). LATS2 activity can be modulated by O-GlcNAcylation in response to high extracellular glucose in MDA-MD-231 cells [41]. Mechanistic studies in HEK293a cells demonstrated that O-GlcNAcylation of LATS2 at T436 disrupts its interaction with MOB1 resulting in LATS2 inhibition and increased activation of YAP/TAZ-TEAD transcription programs [41]. O-GlcNAcylation of Hippo components appears to be quite isoform-specific as it has been detected on MST1 and LATS2, but not MST2 and LATS1 in HEK293 cells [41]. In contrast to O-GlcNAcylation of LATS2, the functional effects of MST1 O-GlcNAcylation are not presently known, as the modification does not affect MST1 phosphorylation/activation in MDA-MB-231 cells [41]. Conversely, in primary mouse hepatocytes and HepG2 hepatocarcinoma cells, LATS1 but not LATS2 undergoes SUMOylation at K830 in response to high cell density, with this modification being required for LATS1 activation and phosphorylation by MST2 [42]. Mechanistic studies in HEK293T cells demonstrated that SUMOylation of LATS1 at K830 increases phosphorylation by MST2 by enhancing interaction between phosphorylated MOB1 and phosphorylated LATS1 and is also able to mask the LATS1-inhibitory effect of SUMOylation at K751 [42], which was demonstrated in a previous study in hepatic cells [43].

### Regulation of Hippo effectors YAP and TAZ

The downstream output of Hippo signaling is mediated by the transcriptional activity of its effectors YAP and TAZ, whose function is tightly regulated through various PTMs (Fig. 2B) that modulate their interaction with transcription factor binding partners, subcellular localization, and proteasomal degradation. Importantly, *activation* of the Hippo pathway kinases ultimately results in *decreased* function of YAP/TAZ by promoting their nuclear exclusion and degradation. In the nucleus, YAP/TAZ can trigger either pro-survival transcription programs through interaction with TEAD [29, 33, 44] or pro-apoptotic transcription through interaction with p73 [45–49], though the latter appears specific to YAP. Two phosphorylation sites have been reported to regulate the switch between these two opposing effects on cell fate: (1) Plk1-mediated phosphorylation of YAP at T77, which enhances affinity for p73 [9]; and (2) c-Abl-mediated phosphorylation of YAP1 at Y357, which inhibits activation of TEAD transcription [50] and promotes interaction with p73 [51]. Notably, unlike the c-Abl-mediated switch which was demonstrated in various non-neuronal cell lines including MCF10A, HEK293, H1299, and HeLa [50, 51], the Plk1-mediated switch has been shown to occur in primary neurons [9]. As Plk1 expression is highest in adult tissues with actively proliferating cell populations and undetectable in normal brain tissues [52], this suggests that in post-mitotic neurons where Plk1 is inactive, nuclear YAP preferentially interacts with TEAD [9]. Functionally, decreased YAP-TEAD interaction in cortical neurons is associated with transcriptional repression induced atypical death of neurons (TRIAD), a form of slow necrotic cell death, which is distinct from apoptosis [8]. Additionally, VGLL4, a key regulator of the hypoxia-sensing pathway [53], competes with YAP for binding to TEAD, thereby antagonizing YAP-TEAD transcriptional activity [29, 54, 55]. However, VGLL4 repression of YAP-TEAD transcription has yet to be demonstrated in brain tissues.

Canonically, Hippo signaling inactivates YAP/TAZ by altering their subcellular localization through LATS1/2-mediated phosphorylation at S127/S89, which promotes YAP interaction with cytosolic 14-3-3 proteins and their subsequent degradation [31, 33, 45, 56, 57]. Thus, LATS1/2-mediated phosphorylation of YAP suppresses interaction with transcription factors including both p73 and TEAD [9]. Interestingly, YAP S127 may also be phosphorylated by Akt, similarly leading to cytoplasmic retention and decreased pro-apoptotic p73 transcription [45], although this PTM appears to be quite cell-type specific as it has been observed in hTert-RPE, Cos-7, MCF7, MDA-468, and H1299 cells [45] but not HeLa or HEK293T cells [33]. LATS1/2 also phosphorylates YAP at S381 [58] and TAZ at S311 [59], leading to further phosphorylation by CK1 and recruitment of β-TrCP1 E3 ubiquitin ligase resulting in YAP/TAZ ubiquitination and degradation. In addition to classical Hippo-dependent phosphorylation, Hippo kinase independent pathways in both the cytoplasm and nucleus further modulate the localization and function of YAP/TAZ through a complex interplay of PTMs, protein interactions, and mechanical signals (reviewed in [28, 60]). Indeed, recent models suggest a continuous shuttling of YAP/TAZ with nuclear import/export rates altered by PTMs, rather than a simple binary switch between cytoplasmic retention and nuclear translocation driven by phosphorylation [61].

Beyond phosphorylation, regulatory PTMs reported for YAP include acetylation, methylation, O-GlcNAcylation, and SUMOylation, though similar work remains sparse for TAZ. In HepG2 hepatocellular carcinoma cells, P300/CBP acetylates, while SIRT1 deacetylates YAP, with deacetylation promoting YAP nuclear localization and interaction with TEAD4 [62]. Methylation at K494 of YAP by SETD7 is required for YAP cytoplasmic retention in response to cell-cell contact in mouse embryonic fibroblasts and impairment of this PTM induces YAP nuclear translocation without influencing its phosphorylation or degradation [63]. It has not yet been elucidated whether phosphorylation of YAP by LATS1/2 is required for SETD7-dependent methylation of YAP. O-GlcNAcylation of YAP at S109 in HEK293T and pancreatic cancer L3.6 cells [64] and at T241 in liver cancer cell lines [65] in response to high extracellular glucose enhances YAP activity and stability by disrupting its interaction with LATS1 and β-TrCP E3 ubiquitin ligase. SUMOylation of YAP1 occurs in response to verteporfin treatment of an endometrial cancer cell line and is regulated by YAP phosphorylation [66]. TEAD1 can also be modified by SUMOylation at K173, which decreases TEAD1 interaction with YAP1 and TAZ, and is involved in oxidative stress during cardiomyocyte hypertrophy [67].

Direct protein-protein interactions can also regulate YAP/TAZ transcriptional activity. For example, in MDCK cells, YAP/TAZ activity can be inhibited by direct binding to AMOTL2 which sequesters YAP/TAZ to tight junctions [68]. In HEK293T and MCF10A cells, YAP/TAZ interaction with TEAD is precluded by SWI/SNF interaction with YAP/TAZ under conditions of low mechanical stress; high mechanical stress stimulates nuclear F-actin accumulation and binding to SWI/SNF, allowing for YAP/TAZ-TEAD interaction [69]. By themselves, mechanical forces, such as cellular stretching and extracellular matrix (ECM) stiffness, can also promote YAP/TAZ nuclear localization in mammary MCF10A and MDA-MB-231 cells, and bone-marrow-derived MSC and HMVEC cells [70]. Furthermore, loss of epithelial tissue organization can release YAP/TAZ from membrane-associated complexes, leading to their nuclear translocation [44, 71, 72]. Together, these diverse regulatory inputs underscore the intricate control of YAP/TAZ localization and activity.

An additional layer of regulatory complexity in mammalian Hippo signaling is achieved by the splitting of the Hippo signaling role of Drosophila *Yki* into two main effectors in vertebrates: YAP and TAZ. Although often treated as functionally redundant, this is only partially true, as these two paralogs have distinct structures, expression, regulatory mechanisms, and interaction partners [44]. Structural differences include the presence of a second phosphodegron on TAZ but not YAP that can be phosphorylated by GSK3α [73], which may explain the lower stability and greater influence of degradation mechanisms on TAZ [44, 59, 74]. Functional divergence is found in HEK293 cells, where deletion of YAP, but not TAZ, impairs cellular glucose uptake and inhibits cell proliferation [75], aligning with studies demonstrating that TAZ does not undergo O-GlcNAcylation in response to changing cellular energy status [64]. In non-small cell lung carcinoma cells, YAP regulates cell cycle progression while TAZ regulates cell migration [76]. Moreover, YAP and TAZ have been found to have differing importance in tumor progression depending on tumor genetic background and cell and tissue contexts [44]. Importantly, such functional divergence may also occur in the nervous system, especially given their cell-type specific distribution, with TAZ transcripts being ubiquitously expressed and YAP transcripts found mainly in astrocytes and endothelial cells [7]. Indeed Mao et al. [9] found that YAP but not TAZ was able to suppress necrotic cell death in a primary cortical neuron model of Huntington’s disease.

Intriguingly, despite the lower neuronal abundance of YAP transcripts, neuronal specific YAP isoforms (YAPdeltaCs) have been described in the brain [8] and spinal cord [10]. YAPdeltaCs contain nucleotide insertions between exons 5 and 6 that lead to frameshifts and C-terminal truncated proteins [8] that lack the transactivation domain [44]. Despite the lack of the C-terminal PDZ-binding motif required for nuclear translocation of full-length YAP in serum-starved HEK293 cells [77], YAPdeltaC immunoreactivity has been demonstrated in the neuronal nuclei [8, 10], suggesting either that the PDZ-binding motif is not necessary for YAP nuclear import in neuronal cells or that YAPdeltaCs are imported via different mechanisms versus full-length YAP. YAPdeltaCs act as pro-survival factors by blocking interaction between full-length YAP and pro-apoptotic p73 [8, 10]. Similarly, alternative isoforms with different signal transduction abilities have been described for TAZ. cTAZ is a truncated isoform of TAZ produced by alternative promoter usage that has been detected in HEK293a cells along with various colon, uveal, lung, and renal cancer cell lines [78]. This truncated isoform does not respond to Hippo regulation and instead suppresses JAK-STAT signaling by direct interaction with STAT1 in colon cancer cells [78].

Overall, YAP and TAZ exhibit differential regulation and function, influenced by both canonical Hippo signaling and non-canonical pathways. Further understanding these distinctions, especially in the context of subcellular localization, isoform usage, and mechanical signaling, particularly within the nervous system will be necessary to elucidate their roles in AD pathogenesis.

### The role of Hippo signaling in the adult brain

While best-known for its role in controlling cell growth and tissue organization, Hippo signaling also plays important roles in the nervous system, being involved in neuronal differentiation, neuroinflammation, neurodegeneration, and neuronal death [7], as well as cell quality control by promoting apoptosis in damaged cells [12]. Hippo signaling plays a crucial role in various aspects of nervous system development, including the proliferation and differentiation of neuronal progenitors [79, 80], neural crest cell migration and differentiation [81], myelination [82], and dendritic arborization [83]. While the neurodevelopmental role of Hippo signaling is well-studied [25, 84], the continued expression of Hippo pathway components in the adult brain suggests a broader role in maintaining cellular homeostasis and function. Indeed, various adaptor proteins which provide additional regulatory complexity by influencing the spatial associations between the core kinases have been found to play additional roles in synaptic structure and neuronal communication. Willin/FRMD6 is an upstream regulator of Hippo signaling that demonstrates differing effects on downstream components depending on cell-context (reviewed in [26]). Specifically, in epithelial cell lines [85] and fibroblasts [86, 87], Willin/FRMD6 acting through Hippo kinases negatively regulates YAP/TAZ, while it positively regulates YAP/TAZ in neuronal [88] and endothelial [89] cells. In fibroblasts, Willin/FRMD6 has recently been shown to interact with MST1/2, leading to YAP/TAZ phosphorylation [87]. In neuronal cells, Willin/FRMD6 is involved in neuronal differentiation [88] and mitochondrial function [90]. Notably, *Frmd6* transcripts are enriched in resting dendrites and localization to dendrites increases in response to depolarization [91]. CAPON, also known as NOS1AP, forms a complex with YAP and promotes phosphorylation of LATS1 and YAP, leading to decreased TEAD activity and cell proliferation [92]. CAPON also modulates glutamate synaptic transmission through interactions with PSD95 and PSD93, which impacts the association of neuronal nitric oxide synthase with PSD95/NMDA receptor complexes [93]. Single nucleotide polymorphisms (SNPs) in *WWC1*, whose protein product KIBRA functions as a scaffold to promote MST1/2 phosphorylation of LATS1/2 [38], have been associated with differences in episodic memory performance, gray and white matter volume, and functional brain activity [94–100]. KIBRA also plays a role in hippocampal-dependent learning and memory by functioning as a scaffold for AMPAR complexes in the postsynaptic compartment [101]. In addition, several GWAS studies have uncovered *WWC1*-associated SNPs associated with AD risk [102, 103].

The expression pattern of Hippo pathway components varies across different brain regions and cell types, suggesting highly context-dependent roles. A transcriptomics database of sorted brain cell types from the cerebral cortex [104] demonstrates cell-type specific expression patterns. While MST1/2, SAV1, LATS1/2, and TAZ are highly expressed in all brain cell types, YAP is predominantly expressed in astrocytes and endothelial cells, with lower expression in neurons and microglia [7]. While YAP protein expression appears to be minimal in neurons of the neocortex and spinal cord during both development and adulthood [105, 106], neuron-specific YAPdeltaC isoforms and FL-YAP have been detected in neurons under pathological insult, such as in the spinal cord of transgenic ALS mice [10]. YAPdeltaCs and FL-YAP have also been detected in cortical and striatal neurons from non-diseased mouse and human brains, though at a much lower level [8]. Similarly, TEAD1-4 exhibit cell type specificity in transcript [7] and protein expression patterns [107].

These differences in expression patterns likely reflect the diverse roles Hippo signaling plays in different cell types, ranging from regulating proliferation and differentiation in neural precursor cells to maintaining cellular homeostasis and mediating neurodegeneration in mature neurons and glial cells. Indeed, in a newly proposed model of Hippo signaling, YAP expression tends to be higher in helper cells, driving their proliferation and preventing differentiation, and lower in worker cells [28]. In the context of the nervous system, this paradigm suggests that high YAP levels in neural precursor cells inhibit differentiation into neurons and promote differentiation into astrocytes and ependymal cells [28]. As an example of brain region differences in this paradigm, studies have demonstrated that YAP is required for neocortical, but not hippocampal, astrogliogenesis, although it is found in astrocytes of both brain regions [105].

Studies in the aging brain reveal that Hippo signaling becomes increasingly activated, with activation associated with astrocytic senescence [21], differential localization of KIBRA to AMPAR complexes [16], and neurodegeneration [27]. Similarly, the aged mouse hippocampus demonstrates increased activation of core Hippo kinases, elevated phosphorylation of YAP, and an overall decrease in YAP expression, particularly in astrocytes [21]. Similar aging-associated changes in Hippo signaling are also observed in in vitro primary astrocytic models of senescence [21]. Though it is not required for hippocampal astrogliogenesis [105], YAP does play a role in preventing hippocampal astrocytic senescence through induction of CDK6 expression [21]. Decreased CDK6 expression with YAP depletion in astrocytes along with studies demonstrating that pharmacological inhibition of MST1/2 reversed signs of astrocytic senescence in WT-YAP but not YAP-KO astrocytes [21], suggest that induction of the YAP-TEAD transcriptional program and its downstream target *Cdk6* [108] is critical in preventing premature senescence [21]. Interestingly, knockout of astrocytic YAP also produces biochemical markers of senescence in neurons, but not microglia [21], indicating a potential cross-talk between astrocytes and neurons mediated by Hippo signaling. Indeed, astrocytes play a key role in promoting neuronal survival [109] and astrocytic deletion of YAP can result in neuronal death [105].

### Hippo signaling in AD pathogenesis

Dysregulated Hippo signaling occurs in multiple pathological contexts. Generally, hypoactive Hippo signaling and YAP hyper-function are associated with overproliferation and cancer, while hyperactive Hippo signaling and YAP hypo-function are associated with cell death and neurodegeneration [27]. Hippo pathway activation contributing to neurodegenerative disease progression has been described in disease models of amyotrophic lateral sclerosis (ALS) [110, 111] and in Huntington’s disease (HD) [11]. Studies in a mouse model of ALS show that MST1 knockout leads to increased neuronal viability, delayed symptom onset, and enhanced survival [111], underscoring the deleterious effects of Hippo kinase activation. Similarly, MST1 activation and reduced YAP activity are found in the cortices of HD patients and an HD mouse model [11]. MST1 activation is also seen in the hippocampus but not pre-frontal cortices of a mouse model of depression [112].

Multiple indirect lines of evidence suggest a role for Hippo signaling in AD pathogenesis. Early evidence came from GWAS studies that identified SNPs in upstream Hippo regulators *FRMD6* [113] and *WWC1* [102, 103] that were associated with AD susceptibility and hippocampal volume changes. Subsequent studies indicated altered expression of these upstream regulators in AD brains and AD mouse and cellular models [90, 114, 115]. Another upstream Hippo regulator *crumbs* (*crb*) was found to be neuroprotective in a *Drosophila* eye model of AD with targeted misexpression of human Aβ42 in differentiating retinal neurons [116]. Additionally, pathway analysis of differentially expressed genes in AD brain tissue demonstrated significant enrichment of Hippo signaling-associated transcripts in the entorhinal cortex [20]. Subsequent co-expression network analysis indicated that YAP was a key hub gene associated with dysregulation of astrocyte-expressed genes in AD brains [20]. Finally, studies in HEK293T cells demonstrate that YAP/TAZ is capable of forming a triple protein complex with Mint3 and the intracellular C-terminal domain of amyloid precursor protein (APP) [117]. As intact APP is a membrane protein, formation of the triple protein complex sequesters YAP/TAZ and Mint3 at the membrane, while APP processing by γ-secretase releases this transcriptionally active complex, allowing for nuclear translocation and transcription of target genes [117]. Transcriptional activation by the complex was demonstrated using an artificial promoter in a luciferase reporter assay, thus the identity of the physiological target genes of the APP-Mint3-YAP/TAZ complex and whether they belong to pro-survival or pro-apoptotic programs is unknown.

**Fig. 3:**
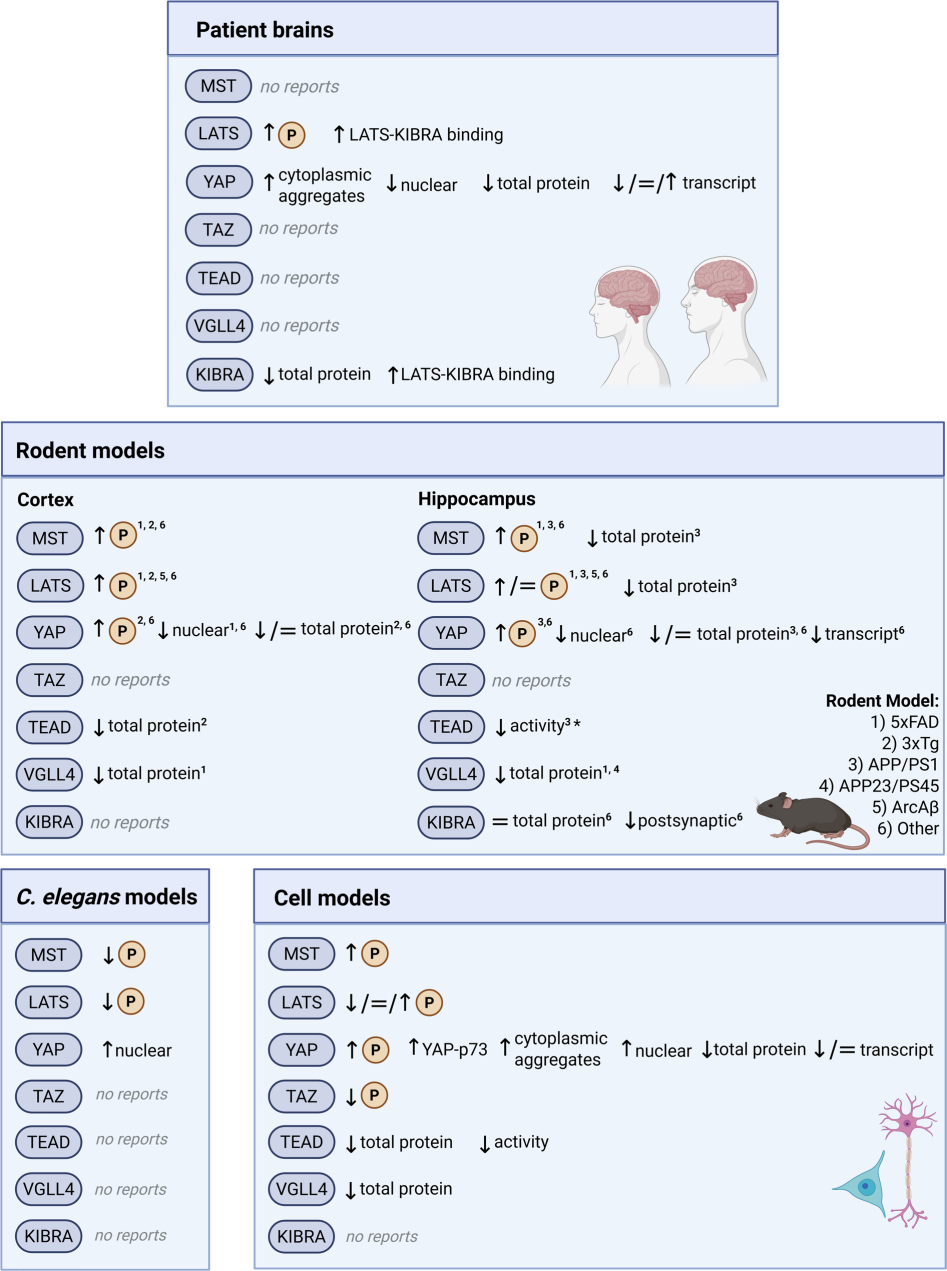
Hippo pathway dysregulation in AD patients and models. AD brains demonstrate increased LATS phosphorylation and increased interaction between LATS and KIBRA [16, 17], along with decreased total YAP protein levels and increased cytoplasmic localization [17], indicating an overall increase in Hippo signaling. However, *YAP* transcript levels demonstrate variable changes depending on disease stage and brain region [20]. KIBRA protein levels are also decreased in AD brain tissue [114, 121]. AD rodent models exhibit increased phosphorylation of MST, LATS, and YAP, alongside overall reduced (or unchanged) YAP protein levels [15–17, 19, 21, 22, 24, 120], and reduced TEAD levels and activity [21, 24] in the cortex and hippocampus. VGLL4 protein levels are also decreased in both the cortex and hippocampus [18], which may phenocopy hypo-activated Hippo signaling due to diminished antagonism of YAP-TEAD signaling. In a tauopathy model based on tau hyperacetylation (tauKQ), total KIBRA levels are unchanged in the hippocampus, but decreased in the postsynaptic compartment [114], suggesting that while tauopathy alone may be insufficient to promote overall decreases, it may promote compartment-specific decreases. In contrast, *C. elegans* AD models show reduced MST and LATS phosphorylation, with increased nuclear YAP [25], indicating reduced Hippo signaling. In vitro models show more varied results. Aβ treatment consistently increases MST phosphorylation [19, 24, 119]. However, LATS phosphorylation can either be increased [24], decreased [25], or unaffected [19] in Aβ-treated cells. Typically, in cell models, alterations in YAP reflect activated Hippo signaling. YAP phosphorylation is increased [24], with reduced total protein [14, 24] and transcript levels [20], and increased cytoplasmic sequestration [17]; however, increased nuclear YAP has been described in cell models subjected to higher Aβ concentrations [23]. TAZ phosphorylation is also reduced under high Aβ concentrations [23]. TEAD protein levels [24], TEAD activity [17], and VGLL4 protein levels [18] are also reduced in in vitro models. = indicates levels unchanged. * indicates change inferred based on downstream targets

As the studies discussed above suggest that Hippo signaling alterations represent an important underlying mechanism in AD pathogenesis, we next describe several recent studies that have provided direct evidence linking Hippo signaling to AD. These studies have further strengthened the connection between Hippo signaling and AD by directly examining dysregulation of core Hippo pathway components in AD brains and related models (Fig. 3; Table 1). Contradictorily, such studies have associated both increased [14–17, 19, 21, 22, 118–120] and decreased [23, 25] Hippo signaling with AD pathogenesis, suggesting biphasic or context-specific dysregulation.

**Table 1:** Hippo pathway dysregulation in AD patients and models

		AD-associated alteration	Outcome
AD brains	LATS	↑ phosphorylation in temporal, occipital [17], and frontal cortices [16]	Hippo activation
		↑ LATS1/2-KIBRA binding in frontal cortex [16]	Hippo activation
	YAP	↓ total and neuronal nuclear protein levels in temporal and occipital cortices [17]	Hippo activation
		↑ cytoplasmic aggregation in temporal and occipital cortices [17]	Hippo activation
		Trend towards ↓ transcripts in incipient AD [20]	Hippo activation
		↑ transcripts in entorhinal cortex, hippocampus, temporal cortex in severe AD [20]	Hypo-Hippo
		No change in transcripts in frontal cortex [20]	No change
	KIBRA	↓ total protein levels [114, 121]	Hypo-Hippo
		↑ LATS1/2-KIBRA binding in frontal cortex [16]	Hippo activation
AD rodent models	MST	↑ phosphorylation in 3xTg cortex [24], 5xFAD hippocampus and cortex [19, 120], APP/PS1 hippocampus [21, 22], ICV-STZ AD rat hippocampus and cortex [15]	Hippo activation
		↓ total protein levels in APP/PS1 hippocampus [21]	Hypo-Hippo?
	LATS	↑ phosphorylation in 5xFAD and APP-KI occipital cortices (3 months) [17], 3xTg cortex [24], APP/PS1 hippocampus [21], male ArcAβ brain [16], ICV-STZ AD rat hippocampus and cortex [15]	Hippo activation
		No change in phosphorylation in 5xFAD hippocampus (7 months) [19]	No change
		↓ total protein levels in APP/PS1 hippocampus [21]	Hypo-Hippo?
	YAP	↑ phosphorylation in 3xTg cortex [24], APP/PS1 hippocampus [21, 22], ICV-STZ AD rat hippocampus and cortex [15]	Hippo activation
		↓ nuclear protein levels in 5xFAD and APP-KI occipital cortex neurons [17] and ICV-STZ AD rat hippocampus and cortex [15]	Hippo activation
		↓ total levels in 3xTg cortex [24] and APP/PS1 hippocampus [21]	Hippo activation
		No change in total protein levels in ICV-STZ AD rat hippocampus and cortex [15]	No change
		↓ transcripts in hippocampus of TAS10/TPM and P301L mice [20]	Hippo activation
	TEAD	↓ activity in APP/PS1 hippocampus [21]*	Hippo activation
		↓ TEAD2 protein in 3xTg cortex [24]	Hippo activation
	VGLL4	↓ protein in neurons and astrocytes but not microglia of APP23/PS45 hippocampus, 5xFAD hippocampus, and 5xFAD temporal cortex [18]	Hypo-Hippo?
	KIBRA	↓ protein in postsynaptic compartment of tauKQ hippocampus [114]	Hypo-Hippo
		No change in total levels in tauKQ hippocampus [114]	No change
*C. elegans* models	MST	↓ phosphorylation in *C. elegans* with Aβ42 overexpression [25]	Hypo-Hippo
	LATS	↓ phosphorylation in *C. elegans* with Aβ42 overexpression [25]	Hypo-Hippo
	YAP	↑ nuclear protein levels in *C. elegans* with Aβ42 overexpression [25]	Hypo-Hippo
Cell models	MST	↑ phosphorylation in PC12 + 2.5-10 µM Aβ [19], PC12 + 8 µM Aβ [24], and in primary rat cortical neurons + 1.5 µM Aβ [119]*	Hippo activation
	LATS	↓ phosphorylation in SH-SY5Y + 25 µM Aβ [25]	Hypo-Hippo
		No change in phosphorylation in PC12 + 2.5–10 µM Aβ [19]	No change
		↑ phosphorylation in PC12 + 8 µM Aβ [24]	Hippo activation
	YAP	↑ phosphorylation in PC12 + 8 µM Aβ [24]	Hippo activation
		↓ total protein levels in PC12 + 8 µM Aβ [24], and BV2 microglial cells + 5-10 µM Aβ [14]	Hippo activation
		↑ aggregates in cytoplasm and ballooned ER of iPSC-derived neurons with SwAPP mutations [17]	Hippo activation
		↑ nuclear translocation in differentiated PC12 + 25 µM Aβ, primary rat hippocampal neurons + 25 µM Aβ, and HEK293T + 25 µM Aβ [23]	Hypo-Hippo
		↑ interaction with p73 in differentiated PC12 + 25 µM Aβ and HEK293T + 25 µM Aβ [23]	Hypo-Hippo
		↓ transcripts in neonatal astrocytes cultured on APP/PS1 brain slices [20]	Hippo activation
		No change in transcripts in adult astrocytes cultured on APP/PS1 brain slices [20]	No change
	TAZ	↓ phosphorylation in SH-SY5Y + 25 µM Aβ [25]	Hypo-Hippo
	TEAD	↓ activity in iPSC-derived neurons with SwAPP mutations [17]	Hippo activation
		↓ total TEAD2 protein levels in PC12 + 8 µM Aβ [24]	Hippo activation
	VGLL4	↓ total protein levels in HEK293T + SwAPP + BACE1 and SHSY5Y + SwAPP [18]	Hypo-Hippo?

*Change inferred based on downstream targets

3xTg – APP Swedish (KM670/671NL), MAPT P301L, PSEN1 M146V. 5xFAD – APP Swedish (KM670/671NL), APP Florida (I716V), APP London (V717I), PSEN M146L, PSEN1 L286V. APP-KI – APP Swedish (KM670/671NL), APP Arctic (E693G), APP Iberian (I716F). APP/PS1 – APP Swedish (KM670/671NL), PSEN1de9. ArcAβ – APP Swedish (KM670/671NL), APP Arctic (E693G). TAS10/TPM – APP Swedish (KM670/671NL), PSEN1 M146V. P301L – MAPT P301L. APP23/PS45 – APP Swedish (KM670/671NL), PSEN1 G384A. tauKQ – hyperacetylation of MAPT lysines K274 and K281. ER – endoplasmic reticulum. SwAPP – APP Swedish (KM670/671NL). ICV-STZ – intracerebroventricular-streptozotocin induced model

A *Drosophila* model also demonstrates general Hippo activation in response to Aβ42 based on genetic depletion of *hpo*,* wts*,* yki* studies and downstream Hippo reporter assays [118]

? Decreased total MST1 and LATS protein levels may indicate hypo-Hippo signaling; however, these changes occurred simultaneously with an increase in phosphorylation of these two kinases, suggesting instead Hippo activation. Decreased VGLL4 levels may phenocopy hypo-Hippo signaling through loss of antagonism for TEAD, thus increasing YAP-TEAD binding; however, this has not been directly assessed

#### Hyper-Hippo signaling in AD

A number of studies suggest that hyperactivation of the Hippo pathway – characterized by increased MST1/2 and LATS1/2 activation, decreased YAP levels, and increased YAP phosphorylation and nuclear exclusion – occurs in AD. For example, LATS1 phosphorylation is significantly increased in AD and mild cognitive impairment (MCI) occipital and temporal cortices [17], as well as in AD frontal cortices [16]. Consistently, AD and MCI cortices demonstrate decreased total and neuronal nuclear YAP protein levels with increased YAP cytoplasmic aggregation in neurons particularly in MCI samples [17]. Similarly, multiple AD rodent models demonstrate protein level changes consistent with Hippo activation and associated with neuronal toxicity in AD-relevant brain regions. These include alterations in the hippocampus [19, 120] and cortex [17] of 5xFAD mice, the cortex of APP-KI mice [17], the cortex of 3xTg mice [24], the hippocampus of APP/PS1 mice [21, 22], the brains of ArcAβ mice [16], and the hippocampus and cortex of an STZ-induced rat model of AD [15].

Activation of the full complement of the classical kinase cascade has been observed in the APP/PS1 hippocampus, in 12-month-old 3xTg cortices, and in neuronal PC12 cells treated with Aβ (8 µM), as evidenced by increased phosphorylation of core Hippo components MST1/2, LATS1/2, and YAP [21, 22, 24], along with decreased overall YAP levels [21, 24]. Reduced TEAD2 levels in 3xTg cortices and Aβ-treated PC12 cells [24], along with reduced expression in the APP/PS1 hippocampus [21] of YAP-TEAD target CDK6 [108], further suggest the involvement of Hippo/YAP-dependent signaling in AD. Interestingly, the APP/PS1 hippocampus also demonstrates increased levels of c-Abl [22], whose phosphorylation of YAP increases pro-apoptotic YAP-p73 interaction [51]. Taken together, these studies suggest suppression of YAP-TEAD transcriptional activity via Hippo kinase activation in both the hippocampus and cortex of AD mouse models and in Aβ-treated PC12 cells. Notably, Hippo activation is associated with cognitive deficits and neuronal loss, as HDAC3 knockdown in the APP/PS1 hippocampus both reverses pathological activation of Hippo signaling and protects against cognitive deficits and neuronal loss [22], implying an upstream role for HDAC3 in Hippo pathway regulation, though whether the cognitive improvements directly resulted from inactivation of Hippo signaling was not fully explored in the study. Although the specific cell types affected were not directly examined, HDAC3 is predominantly expressed in neurons, indicating that neuronal Hippo activation contributes to AD pathogenesis [22].

Genetic manipulation studies in a *Drosophila* model of AD provide direct evidence that Hippo activation contributes to neuronal Aβ toxicity. Hippo activation as a result of expression of human Aβ42 in differentiating retinal neurons in this model is evidenced by downregulation of downstream reporters diap1-4.3-GFP and ex-lacZ [118], and enhanced JNK signaling [118, 122], both of which were associated with increased neuronal death. Importantly, blockade of Hippo signaling by knockdown of MST1/2 or LATS1/2 homologs or expression of YAP homolog ameliorated Aβ-induced cell death, while expression of MST1/2 homolog enhanced the neurodegenerative phenotype, demonstrating the direct involvement of Hippo signaling in Aβ toxicity [118]. Similarly, in mammalian AD models, the direct contribution of MST1 activation and YAP inhibition to AD pathology has been demonstrated through studies involving genetic and pharmacological manipulation of Hippo components (Table 2). However, no corresponding studies have been performed for mammalian LATS1/2, indicating an important area for future investigations.

**Table 2:** Hippo pathway manipulation in in vivo mammalian AD models

AD Model	Hippo manipulation	Outcomes	Reference
5xFAD (3-5 month)	MST1 overexpression (AAV)	Accelerated cognitive impairment, LTP impairment, synaptic and neuronal loss, neuronal apoptosis, mitochondrial dysfunction and oxidative stress. No change in Aβ levels.	[19, 120]
5xFAD (7-8 month)	MST1 knockdown (AAV)	Rescued cognitive decline and LTP impairment; reduced neuronal apoptosis, synaptic damage, mitochondrial dysfunction and oxidative stress	[19, 120]
5xFAD (8 month)	MST1 pharmacological inhibition (XMU-MP-1)	Rescued cognitive decline, synaptic impairment, synaptic loss; reduced neuronal apoptosis; improved mitochondrial homeostasis; PI3K-Akt activation	[19, 120]
APP/PS1 (6 month)	MST1 pharmacological inhibition (XMU-MP-1)	Delayed astrocytic senescence, rescued cognitive decline	[21]
ICV-STZ rats	MST1 pharmacological inhibition (XMU-MP-1)	Rescued cognitive decline; decreased tau hyperphosphorylation, amyloid plaques, oxidative stress, acetylcholinesterase hyperactivity, neuroinflammation, synaptic dysfunction, neuronal apoptosis, neuronal loss	[15]
Male ArcAβ (9-13 month)	MST1 pharmacological inhibition (XMU-MP-1)	Rescued cognitive decline	[16]
5xFAD (6 month)	YAPdeltaC (AAV)	Rescued cognitive decline, stabilized ER, decreased extracellular but not intracellular Aβ	[17]
5xFAD (6 month)	YAP pharmacological activation (S1P)	Rescued cognitive decline, stabilized ER, decreased extracellular but not intracellular Aβ	[17]
tauKQ * (10-18 month)	CT-KIBRA (lentivirus)	Restored hippocampal LTP, rescued cognitive decline. No effect on tau phosphorylation or CA1 synaptic loss	[121]

*The role of KIBRA with regards to Hippo regulation in AD is not fully understood. This study in tauKQ mice demonstrates that KIBRA rescued AD pathology through a Hippo-independent mechanism

Further investigations indicate that Aβ can promote cell death through both Hippo/YAP-dependent [17] and Hippo/YAP-independent pathways [19, 119]. Loss of YAP-TEAD activity through increased Hippo kinase activation is associated with a form of neuronal necrosis – termed transcriptional repression-induced atypical cell death (TRIAD) – that has recently been identified as a major mechanism of neuronal loss in early AD pathogenesis [17]. TRIAD is marked by ER enlargement and instability and can be induced by inhibition of RNA polymerase II [8, 13, 123] or disruption of nuclear YAP-TEAD interaction [9, 17]. While TRIAD occurs throughout AD pathogenesis, this form of neuronal death is particularly prevalent in MCI patients and in AD mouse models prior to the onset of cognitive impairment [17]. In cortical neurons of both AD and MCI patient brains, as well as 5xFAD and APP-KI AD mouse models, decreased nuclear YAP levels are associated with necrosis, with YAP mislocalization driven by intracellular Aβ sequestration of YAP into cytoplasmic aggregates [17]. These brain tissues also displayed increased neuronal staining of pLATS-S909, with a larger increase in MCI versus AD brains [17], further suggesting that Hippo activation contributes to early AD pathogenesis. Complementary time-lapse studies in human iPSC-derived neurons carrying the APP KM670/671NL mutation and AD mice provide further evidence of the pathological sequence of events with intracellular Aβ sequestering YAP in the cytoplasm, leading to decreased YAP-TEAD transcriptional activity, and subsequent ER ballooning and cell death [17]. Importantly, in vivo injection of AAV-YAPdeltaC, a neuron-specific YAP isoform, protected against cognitive decline and increased extracellular Aβ and ER instability without changing intracellular Aβ levels in 5xFAD and APP-KI mice [17], demonstrating that YAP is involved in Aβ-induced neurotoxicity and cognitive decline. This also indicates the therapeutic potential of increasing neuronal YAP levels, and its downstream TEAD-mediated transcriptional program, particularly at early stages of the disease. Intriguingly, only a fraction of neurons possessing intracellular Aβ undergo TRIAD necrosis [17], suggesting that surviving neurons are lost through other mechanisms.

These alternative mechanisms may involve Hippo/YAP-independent MST1-mediated neuronal apoptosis. Studies in 5xFAD mice and neuronal cell lines demonstrate that MST1 can promote AD pathology and neuronal loss independently of canonical Hippo/YAP signaling by promoting phosphorylation/activation of pro-apoptotic p53 [19] or inhibiting PI3K-Akt signaling [120]. Specifically, Aβ-treatment of neuronal PC12 cells or SH-SY5Y cells led to dose-dependent increases in MST1 activation, resulting in increased apoptosis as measured by expression of apoptosis-related proteins (Bcl2, Bax, cleaved caspase 3, and cytochrome c), which was reversed by genetic knockdown of p53 [19] or pharmacological activation of PI3K-Akt [120]. Similar disease-progression-dependent increases in MST1 activation associated with increased apoptosis have been described in the 5xFAD hippocampus [19, 120], along with increased MST1 activation in the 5xFAD cortex [120]. Furthermore, despite increased MST1/2 activation, phosphorylation of LATS1/2 at T1079/1041 was not increased in either the Aβ-treated PC12 cells or the 5xFAD mouse hippocampus [19], suggesting a Hippo/YAP-independent mechanism. Lack of increased LATS1/2 phosphorylation in the 7-month-old 5xFAD mouse hippocampus [19] contrasts with the previous finding of increased LATS1/2 phosphorylation in the 3-month-old 5xFAD mouse cortex [17], suggesting potential regional and temporal differences in Hippo-related dysfunction in AD. Studies using genetic and pharmacological manipulation of MST1 levels in in vitro and in vivo models further demonstrated the direct contribution of MST1 to neuronal apoptosis, with overexpression of MST1 accelerating and depletion/inactivation of MST1 rescuing neuronal apoptosis [19, 120]. The finding of MST1-mediated pro-apoptotic activation of p53 [19] expands upon previous studies demonstrating that p53 activation contributes to neuronal death in AD [124] and that p53 and Hippo pathways are connected by a complex context-specific regulatory network [125].

Similarly, another study demonstrated that MST1 can mediate neuronal death through activation of FoxO3a [119]. Aβ oligomers (1.5 µM) treatment of primary rat cortical neurons led to increased phosphorylation of FoxO3a at S207 [119], a PTM associated with MST1 activation in response to oxidative stress [126]. This suggests that Aβ oligomers can activate MST1 in primary cortical neurons, leading to activation and nuclear localization of FoxO3a and subsequent induction of neuronal death [119]. Enhanced nuclear localization of FoxO3a in response to Aβ was further confirmed in vitro in primary hippocampal neurons and differentiated PC12 cells and in vivo in APP/PS1 mice at the plaque-bearing stage and in wild-type rats injected with Aβ [119]; however, changes in phosphorylation of FoxO3a at the MST1-specific site were not assessed in these models, leaving open the possibility that the observed increase in FoxO3a nuclear localization could have been due to phosphorylation by other kinases such as JNK [127], p38 [128], or AMPK [129], which similarly stimulate FoxO3a nuclear localization.

Hippo/YAP-independent pathways may also contribute to synaptic and mitochondrial dysfunction, both of which are key features of early AD [130–136]. A recent study in Aβ-treated SH-SY5Y cells demonstrated that neuronal activation of MST1 mediates mitochondrial dysfunction independent of Hippo/YAP signaling through MST1 interaction with PGC1α, leading to decreased expression of mitochondrial DNA (mtDNA)-encoded genes for respiratory chain subunits, resulting in increased oxidative stress [120]. Another study demonstrated that LATS1/2 affects neuronal function through interactions with KIBRA [16]. Specifically, increased phosphorylation of LATS1/2 was observed in brains from male ArcAβ mice, aged male mice, and AD patients [16]. In vitro and in vivo studies in primary hippocampal neurons, human brain-derived organoids, and male ArcAβ mice employing pharmacological inhibition of MST1/2 demonstrated that MST1/2-induced phosphorylation of LATS1/2 promotes interaction with KIBRA [16]. Consistently, AD patient brains also exhibited increased binding of KIBRA to LATS1/2 and reduced binding of KIBRA to GLUA1, suggesting that LATS1/2 may sequester KIBRA from AMPAR complexes during AD pathogenesis, thereby contributing to synaptic dysfunction [16].

In addition to the role of Hippo activation in neuronal dysfunction and death described above, alterations in Hippo signaling leading to reduced YAP levels may also occur in glial cells, contributing to AD pathogenesis through non-neuronal mechanisms. Ex vivo studies demonstrate that astrocytes exposed to Aβ through culturing on neonatal APP/PS1 brain slices have reduced *Yap1* transcript levels, though this dysregulation does not occur with astrocytes cultured on adult APP/PS1 brain slices [20, 137]. Reduced YAP levels are also observed in astrocytes within the hippocampus of APP/PS1 mice, where they promote premature astrocytic senescence through decreased CDK6 expression [21], which can lead to increased secretion of inflammatory cytokines [138] and other senescence-associated secretory phenotype (SASP) factors that disturb surrounding cells and exacerbate AD pathology [139]. For instance, astrocytic YAP deletion results in increased expression of senescence marker Lamin B1 in neurons of the mouse hippocampus [21]. Similarly, Aβ treatment also reduces total YAP expression in microglial BV2 cells leading to enhanced inflammatory cytokine secretion [14], highlighting the impact of Hippo signaling on glial dysfunction in AD.

**Fig. 4:**
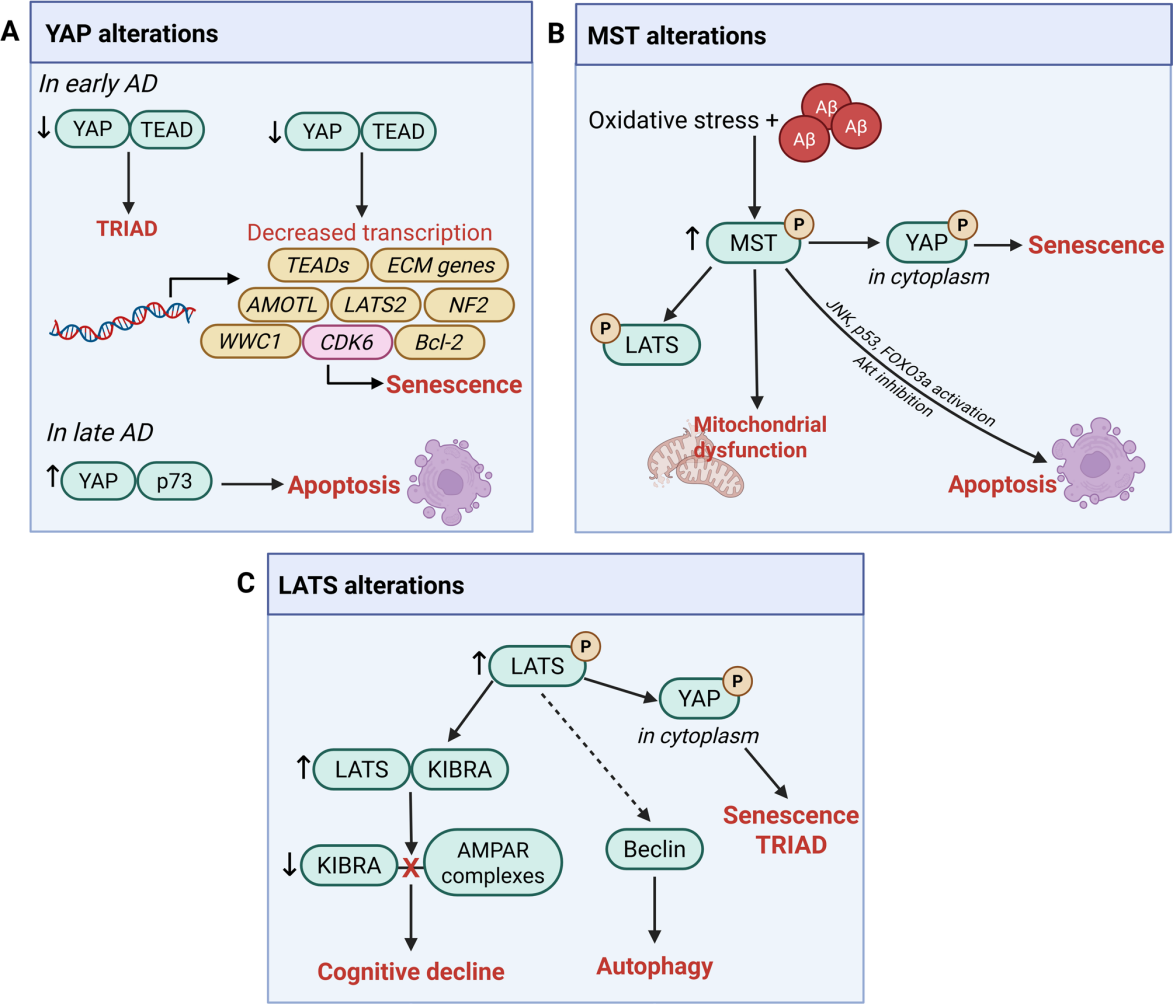
Hippo pathway alterations in Alzheimer’s disease. Dysregulated Hippo signaling can contribute to AD pathogenesis through multiple mechanisms. (**A**) YAP alterations. In early AD, reduced nuclear YAP levels result in decreased YAP-TEAD interactions, leading to transcriptional repression-induced atypical cell death (TRIAD) [17] and diminished expression of YAP-TEAD target genes. Protein products of these include TEADs, AMOTL [68], extracellular matrix (ECM) genes [70], LATS2, NF2 [28], and KIBRA [39]. Reduced expression of cell cycle regulator CDK6 leads to astrocytic, and potentially, neuronal senescence [21]. Under higher Aβ burden, as occurs in late AD, YAP preferentially binds p73 over TEAD, leading to apoptosis [23]. (**B**) MST alterations. Oxidative stress and Aβ accumulation activate MST1/2, leading to increased phosphorylation of LATS, and pro-apoptotic changes including activation of p53 [19], JNK [118, 170], and FOXO3a [119], and inhibition of PI3K-Akt [120]. Increased phosphorylation of MST promotes YAP cytoplasmic retention, leading to cellular senescence via reduced *Cdk6* transcription [21, 108]. YAP sequestration in the cytoplasm can also lead to TRIAD [17], though it is presently unknown whether this process is MST1/2-dependent. MST1/2 activation can also lead to mitochondrial dysfunction [120]. (**C**) LATS alterations. LATS phosphorylation is elevated in AD, leading to increased YAP phosphorylation and nuclear exclusion, resulting in cellular senescence [21] and TRIAD [17]. Increased LATS phosphorylation also enhances interaction with KIBRA, leading to reduced KIBRA availability for AMPAR scaffolding, thereby contributing to synaptic dysfunction and cognitive decline [16]. In addition, though yet to be established in the context of AD, LATS1 but not LATS2, regulates autophagy independently of its kinase function through direct binding to and stabilization of core autophagy protein Beclin [196]. Thus, decreased total levels of LATS protein, which have been observed in AD mice [21], may contribute to autophagic dysfunction.

In summary, hyper-activated Hippo signaling in AD could deleteriously impact the brain through multiple mechanisms (Fig. 4) including enhanced astrocytic senescence, which could promote neuronal senescence [21]; decreased YAP-TEAD transcriptional activation leading to neuronal necrosis [17]; increased neuronal apoptosis through MST1/2-mediated activation of p53 [19] and FoxO3a [119] or inhibition of PI3K-Akt [120]; increased mitochondrial dysfunction through MST1/2 disruption of mtDNA transcription/translation [120]; and increased synaptic deficits through LATS1/2 sequestration of KIBRA from AMPAR complexes [16]. Notably, many of these studies examining neuronal Hippo dysfunction have utilized experimental systems involving supra-physiological expression of APP or exogenous exposure to Aβ. Such models could induce non-physiological cellular events and cell death phenotypes, and may therefore demonstrate pathological mechanisms that are of limited relevance to neurons in the human AD brain. In this regard, in vivo and in vitro APP knock-in models, which preserve endogenous regulatory elements and therefore avoid ectopic overexpression, may provide more disease-relevant results and help to distinguish disease-related pathways from artifacts of overexpression systems. As described above, Tanaka et al. [17]. employed such models to demonstrate that YAP-TEAD-dependent neuronal necrosis is an important mechanism of neuronal loss in early AD. Intriguingly, as YAP-TEAD signaling regulates expression of mitochondrial proteins PGC1α and MFN1/2 in HUVE cells [140], it is possible that decreased YAP levels/activity in AD due to hyper-activation of Hippo signaling could also exacerbate mitochondrial dysfunction. In addition, as YAP promotes glycolysis by promoting glucose uptake [141], decreased YAP levels could exacerbate neuronal glucose deficits in AD [142]. However, the exact contribution of these processes to AD pathogenesis requires further elucidation, particularly regarding whether and when these changes occur in neurons or glial cells, and how they might interact across different cell types within the brain.

Overall, these studies suggest that therapeutic targeting of Hippo signaling for AD should aim to *inhibit* Hippo kinases or increase YAP levels; however, two studies in neuronal cell lines and *C. elegans* models present results implying that hypo-Hippo signaling could also exacerbate AD pathology, highlighting the need to clarify the context-dependent nature of Hippo signaling in AD. Below, we describe these two studies and propose a model to unite the seemingly contradictory findings.

#### Hypo-Hippo signaling and the detrimental effects of increased YAP levels

Hypo-Hippo signaling was noted in *C. elegans* models of AD with global or neuronal overexpression of mammalian Aβ42, where reduced phosphorylation of MST1 and LATS1/2 homologs *cst-1* and *wts-1* led to enhanced nuclear translocation of unphosphorylated YAP via interaction with Mint3 homolog *lin-10* [25]. YAP in the nucleus of the *C. elegans* models interacted with TEAD homolog *egl-44* [25]. Furthermore, in the *C. elegans* AD model, decreased Hippo signaling and enhanced YAP nuclear localization were associated with Aβ toxicity, learning deficits, and abnormal lysosomal morphology, which were alleviated by decreasing *yap-1* expression [25]. Notably, in the *C. elegans* model YAP-TEAD interaction was deleterious [25], contrasting with previous findings in other AD model systems demonstrating the pro-survival effects of YAP-TEAD interaction. This discrepancy could be due to the fact that *C. elegans yap-1* may only partially share characteristics with mammalian YAP. For example, *yap-1* localization does not respond to depletion of Hippo pathway members upstream of LATS1/2 and *yap-1* overexpression reduces *C. elegans* lifespan [143]. By contrast, mammalian YAP1 inhibits senescence of human fibroblast cells [108, 144], human mesenchymal stem cells [145], human periodontal ligament stem cells [146], glioma cells [147], and astrocytes [21]. Thus, the results in the *C. elegans* model may have low relevance towards understanding Hippo signaling in AD.

Studies using mammalian cell culture models of AD have also demonstrated hypo-Hippo signaling. While application of Aβ at lower concentrations (2.5–10 µM) to PC12 cells results in Hippo activation consistent with phosphorylation and nuclear exclusion of YAP [19, 24], relatively high concentrations (25 µM) of Aβ25–35 induced nuclear translocation of YAP and enhanced pro-apoptotic interaction with p73 in differentiated neuronal PC12 cells [23]. The higher Aβ concentration used in the study from Zhang et al. [23] may have triggered different cellular responses [148] compared to the lower Aβ concentrations used in other studies [19, 24, 119]. For example, higher Aβ levels may have induced YAP nuclear translocation by upregulating YAP nuclear importers. Aβ treatment of primary rat cortical neurons increases expression of HIF-1α, whose activity is sensitive to differing Aβ levels [149], and which has recently been shown to be a direct carrier for YAP nuclear import [150]. In the nucleus, higher Aβ levels may have induced a switch from pro-survival YAP-TEAD signaling to pro-apoptotic YAP-p73 signaling by activating kinases such as c-Abl [51] or Plk1 [9], both of which can increase YAP affinity for p73. In support of this, studies demonstrate that Plk1 levels [151] and activity [152] are increased in AD brains and that c-Abl is activated in rat hippocampal neurons by Aβ fibrils [153]. Indeed, additional experiments in HEK293T cells demonstrated that 25 µM Aβ25–35 treatment resulted in YAP phosphorylation at Y357 [23], a PTM associated with decreased TEAD activity [50] and increased interaction with pro-apoptotic p73, and which is phosphorylated by c-Abl [51]. Consistently, YAP knockdown in PC12 cells attenuated signs of Aβ-induced apoptosis, including activated bax, and increased levels of puma and cleaved caspase 3 [23].

Interpretation of the findings from Zhang et al. [23]. should take into account several caveats such as the Aβ preparation methodology (aggregation in water for five days), which may have resulted in larger Aβ species, and the use of ectopic expression of fluorescently-tagged proteins for interaction studies, which could produce experimental artifacts. While not directly explored by Zhang et al. [23], the increased nuclear translocation of YAP in neuronal cells in response to Aβ could have been mediated through decreased Hippo signaling. Indeed, in a subsequent study from Zhu et al. [25], neuronal SH-SY5Y cells treated with 25 µM Aβ25–35 displayed decreased phosphorylation of LATS1/2 [25]. Though the mechanism underlying high Aβ-induced decreases in LATS1/2 phosphorylation remains unknown, this could align with reports demonstrating lack of [19] or lessened increase in LATS1/2 phosphorylation [17] at more advanced disease stages in AD brains and mice. Thus, these in vitro studies indicating hypo-Hippo signaling may better reflect the pathological mechanisms that occur at a later stage of AD pathogenesis, when Aβ levels are higher, suggesting that under higher Aβ burden inhibiting Hippo signaling and promoting YAP nuclear translocation could be detrimental.

#### A biphasic model of Hippo dysregulation in AD

**Fig. 5:**
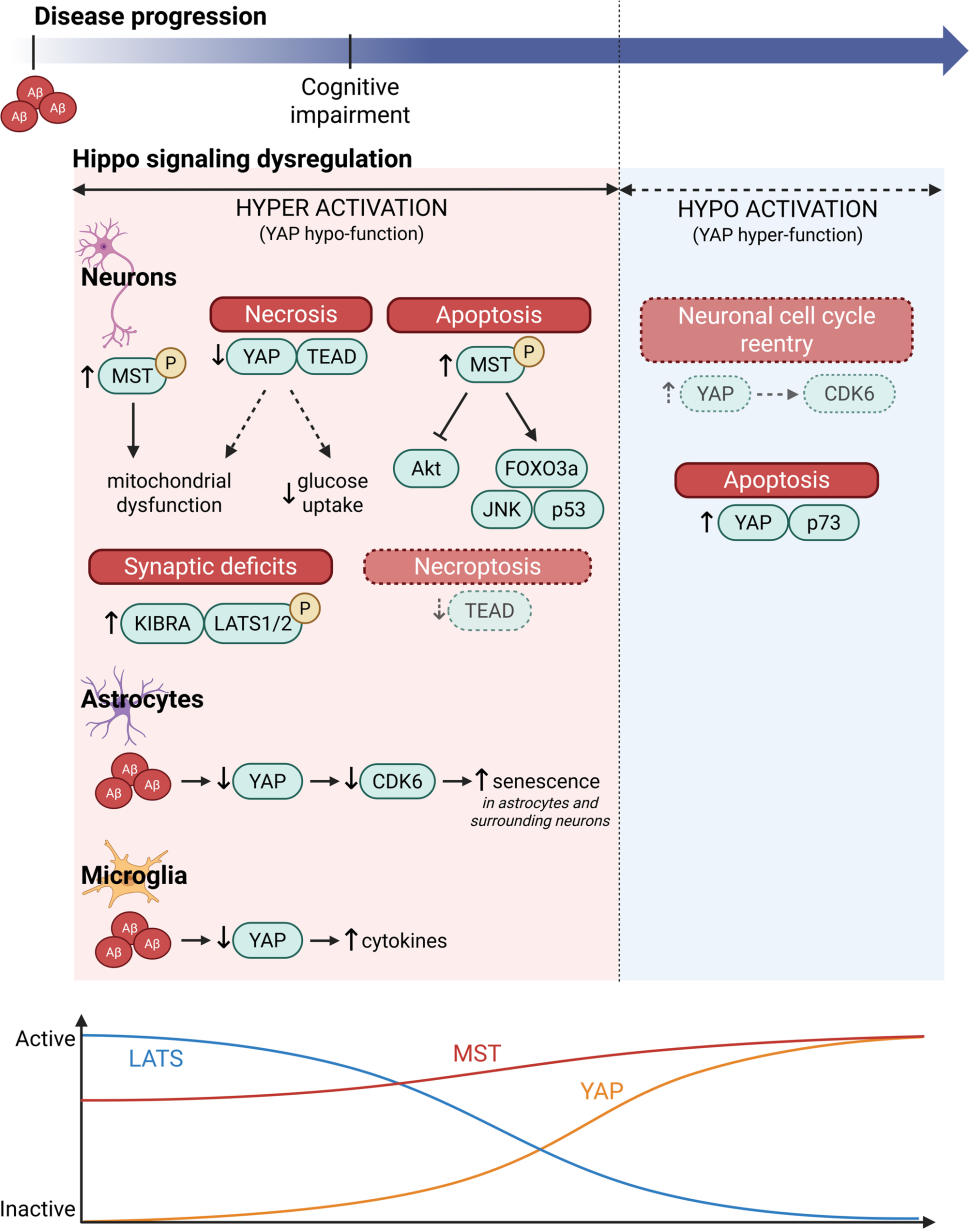
Hypothetical model of Hippo dysregulation during AD pathogenesis. Evidence from AD brains and models suggests a biphasic dysregulation of Hippo signaling, with an early hyper-activated and a later hypo-activated phase, both contributing to neuronal loss via distinct mechanisms. In early AD, Aβ accumulation induces LATS1/2 phosphorylation, increasing binding to KIBRA. This sequesters KIBRA from AMPAR complexes, resulting in synaptic deficits [16]. Intracellular Aβ also sequesters YAP in the cytoplasm, decreasing YAP-TEAD activity and triggering necrosis, which precedes the onset of cognitive decline [17]. Downregulation of YAP-TEAD target genes, including *LATS2* [28], *TEADs* [68], and *WWC1* [39], may contribute towards the shift to the hypo-Hippo stage. Decreased YAP-TEAD activity may also lead to mitochondrial dysfunction [140] and impaired glucose uptake [141], though this has yet to be demonstrated in AD. MST1 activation can also promote mitochondrial dysfunction [120]. Though not yet demonstrated in neurons, TEAD depletion in cardiomyocytes induces necroptosis by disrupting transcription of nuclear-encoded mitochondrial genes [154]. Not all neurons undergo TRIAD [17]; others may succumb to MST1/2-mediated Hippo/YAP-independent apoptosis [19, 118, 119, 170]. Hyper-activation of Hippo in glia may exacerbate neuroinflammation [14] and senescence [21], increasing neuronal injury. In later stages, further Aβ accumulation could trigger the transition toward hypo-Hippo signaling via Aβ-induced activation of c-Abl [153, 240–242] or Plk1 [151, 152], which increases YAP-p73 interaction [9, 50, 51]. In this later phase, YAP-p73-dependent apoptosis is the predominant mechanism underlying neuronal loss. Alternatively, aberrant YAP-TEAD-driven expression of cell cycle regulators like CDK6 may induce cell cycle re-entry and apoptosis in neurons. MST1/2 activation may persist into this later stage, contributing to neuronal toxicity via Hippo-independent pathways. Certainly, AD mouse models suggest continued enhancement of MST1/2 activation with disease progression [19, 120]. To date, evidence for hypo-Hippo signaling has not been found in glia, suggesting that this may be a neuron-specific alteration. These context-specific perturbations may underlie the heterogeneous modes of neuronal death observed in AD [274]

Overall, the studies in mammalian in vitro and in vivo models suggest a putative model (Fig. 5) where hyper-activated Hippo signaling contributes to neuronal dysfunction in early AD, with a potential later stage involving hypo-activated Hippo signaling. During this early stage, hyper-Hippo signaling leads to synaptic deficits through increased sequestration of KIBRA from AMPAR complexes [16] and mitochondrial dysfunction through increased activation of MST1/2 [120]. Additionally, hyper-Hippo signaling may induce neuronal loss through YAP-TEAD-dependent necrosis [17] along with MST1/2-induced apoptosis via p53 [19], FoxO3a [119], and/or suppression of PI3K-Akt [120]. Furthermore, decreased YAP-TEAD activity could lead to decreased TEAD levels as TEAD itself is a downstream transcriptional target [28]. Indeed, decreased TEAD2 levels have been found in the 3xTg mouse cortex and in Aβ-treated PC12 cells [24]. Given that TEAD1 deletion in mice activates cardiomyocyte necroptosis but not apoptosis, through loss of transcription of nuclear-encoded mitochondrial genes essential for ETC assembly [154], it is possible that decreased TEAD levels in AD could also lead to necroptosis. However, it is presently unknown whether this signaling modality reported in cardiomyocytes [154] functions in neurons, especially as mature post-mitotic neurons do not express TEAD1 [107].

Evidence suggests that hyper-activated Hippo signaling precedes cognitive impairment, as it has been noted in MCI brains and in AD mouse models at timepoints prior to the appearance of behavioral deficits [17, 21, 22]. In fact, KIBRA-LATS mediated synaptic deficits [16] may contribute to the emergence of cognitive symptoms. Additionally, there is a potential spatiotemporal element to LATS1/2 activation since the extent of LATS1/2 activation decreases at later stages in patients [17] and since LATS1/2 activation is observed in the 5xFAD mouse model in the cortex at younger ages [17] but not in the hippocampus at older ages [19]. These changes in LATS1/2 activation would support a model where YAP-TEAD-dependent necrosis predominates early on with MST1/2-dependent apoptosis occurring after symptom onset. Recent studies demonstrating increasing MST1 activation with increasing disease progression and apoptosis in the 5xFAD hippocampus [19, 120] further support this model. Hyper-Hippo signaling alterations in the astrocytes and microglia could exacerbate neurodegeneration by promoting neuronal and astrocytic senescence [21] and neuroinflammation [14].

Transcriptomic studies of AD patient brains [155] and AD mouse models [156] demonstrating dynamic changes in *YAP1* transcript levels during the course of AD pathogenesis [20] provide additional support for the biphasic model proposed here. *YAP1* transcripts decrease in incipient AD patients and in the hippocampus of AD mice at an early timepoint prior to the appearance of characteristic pathology, but subsequently increase during later stages [20]. Consistently, in a combined analysis of post-mortem AD patient brain RNA expression datasets, *YAP1* transcripts were found to be significantly upregulated in the entorhinal cortex, hippocampus, and temporal cortex in severe AD [20]. Increased *YAP1* transcripts at later stages would be consistent with hypo-Hippo signaling, though they could also reflect a compensatory return to baseline levels following initial pathological decreases.

Outside of these transcriptomic studies, the putative hypo-Hippo signaling stage has yet to be observed in AD brains or rodent models. Direct evidence for hypo-Hippo signaling is mainly derived from in vitro studies involving exogenous application of high Aβ concentrations to neuronal cell lines, which suggest that hypo-Hippo signaling leads to YAP-p73 dependent neuronal apoptosis [23, 25]. Additionally, while the above discussion attributed the contradictory findings of YAP-TEAD mediated deleterious outcomes in *C. elegans* versus its pro-survival effects in other models to differences in experimental systems, an alternative explanation is that aberrant activation of YAP-TEAD transcriptional programs linked to cell cycle progression in neurons could lead to cell cycle re-entry induced neuronal apoptosis. For example, YAP-TEAD has been shown to drive expression of astrocytic CDK6 [21]. While YAP-TEAD driven expression of CDK6 in neurons has yet to be established, previous studies have demonstrated that induction and activation of CDK6 in neurons leads to cell cycle re-entry resulting in apoptosis, which has been implicated in neuronal loss in AD [157, 158].

If the later hypo-Hippo stage indeed exists, then what might drive the transition between the two stages? We speculate that Aβ accumulation could potentially drive the transition between hyper and hypo-Hippo phases of the disease by altering YAP dynamics. First, Aβ sequestration of YAP could lead to decreased expression of downstream targets of YAP-TEAD transcription that are also upstream Hippo pathway members such as LATS2 [159, 160], and KIBRA [39]. Indeed, decreased total LATS1/2 protein levels are found in the APP/PS1 hippocampus [21] and decreased KIBRA levels have been found in AD brains [114, 121]. Second, Aβ has been show to activate c-Abl [153] and Plk1 [151, 152] kinases, both of which are able to phosphorylate YAP to promote interaction with p73 and subsequently induce YAP-p73 dependent apoptosis [9, 45, 51]. Third, Aβ-mediated increases in expression of HIF-1α [149], a direct importer of YAP [150], could increase YAP nuclear import rates. Lastly, increased APP metabolism [161, 162] could also increase levels of transcriptionally active APP-Mint3-YAP complex in the nucleus by dissociating the complex from the plasma membrane through cleavage of full-length APP [117], though the existence of this complex has yet to be shown in neurons.

#### Dysregulation beyond the core kinase cascade

In addition to core Hippo components, AD pathogenesis may also involve Hippo pathway members that modulate signaling output, adding an additional layer of complexity.

VGLL4, which antagonizes YAP-mediated transcription by competing for TEAD binding [54], is downregulated mainly in the neurons in the hippocampus and temporal cortices of APP23/PS45 and 5xFAD mice [18]. Decreased VGLL4 expression mediates neurotoxicity, as overexpression of VGLL4 in SH-SY5Y cells stably expressing SwAPP695 protects against increased amyloidogenic processing and synaptic protein loss by inhibiting degradation of LDHA and increasing lactate production [18]. Though not yet explored, decreased VGLL4 levels could liberate TEAD, and thus affect Hippo signaling by increasing YAP-TEAD binding, which may lead to aberrant neuronal cell cycle re-entry, although TEAD may bind other transcriptional co-activators in this context.

Upstream Hippo pathway component KIBRA plays a dual role in signal transduction and synaptic function, promoting LATS1/2 phosphorylation through both MST-dependent [38] and -independent mechanisms [39, 40], and serving as a scaffold for AMPAR complex formation [101]. Several lines of evidence point to a role for KIBRA in AD pathogenesis. Initial investigations uncovered SNPs in the *WWC1* gene that were associated with differential memory performance, episodic memory, and AD susceptibility [94, 96, 102, 163–167]. Subsequently, reduced KIBRA expression has been demonstrated in AD brains [114, 121]. Given its functions in scaffolding AMPAR complexes [101] and mediating LATS1/2 phosphorylation [38–40], decreased KIBRA levels in the AD brain may directly compromise synaptic function and Hippo signaling. Indeed, in the hippocampus of an AD mouse model (tauKQ) that mimics the tau hyperacetylation seen in AD brains, KIBRA levels were specifically reduced in the postsynaptic compartment [114]. Reduced levels of postsynaptic KIBRA led to synaptic deficits by impairing AMPAR trafficking [114]. Furthermore, decreased KIBRA levels in AD could reduce LATS1/2 phosphorylation, leading to increased nuclear YAP levels, which could promote neurodegeneration through p73-mediated apoptosis or aberrant cell cycle re-entry through interaction with TEAD, though the link between decreased KIBRA levels and hypo-Hippo signaling has yet to be demonstrated in AD models.

#### Is Hippo dysregulation upstream or downstream of Aβ?

A frequent question in the AD field is how aberrant signaling is related to changes in Aβ. For Hippo signaling, in vivo studies indicate that dysregulation often occurs downstream of Aβ alterations. For example, while AAV-mediated overexpression of MST1 in the hippocampus does not increase Aβ levels in either wild type (WT) or 5xFAD mice [19, 120]; overexpression of MST1 does result in a greater increase in MST1 activation in 5xFAD mice compared to WT mice, suggesting that the presence of Aβ can potentiate Hippo pathway dysregulation [19]. Likewise, while administration of AAV-YAPdeltaC protects against cognitive decline and pathological changes, including increased extracellular Aβ and ER instability, in 5xFAD and APP-KI mice, intracellular Aβ levels remain unchanged [17]. In addition, genetic manipulation of MST1 homolog *hpo* in a *Drosophila* model of AD does not alter Aβ levels [118]. Complementing these genetic manipulation studies, time-lapse imaging in AD mice and cell models demonstrates that the pathological cascade resulting in neuronal necrosis is initiated by intracellular Aβ sequestration of YAP [17].

By contrast, *C. elegans* mutants in *cst-1* and *egl-44*, MST and TEAD homologs, do exhibit altered Aβ levels [25], demonstrating that in this particular model system Hippo signaling may lie upstream of Aβ accumulation. However, as discussed above, mammalian Hippo-YAP signaling functions may not be fully preserved in *C. elegans*. Nevertheless, additional studies in mammalian cells also demonstrate that modulating Hippo activity can affect Aβ production. For example, in alignment with the results demonstrating the association between decreased YAP levels and AD pathology, YAP knockdown in glial U251 cells expressing mutant APP increased levels of Aβ42 and tau phosphorylation, whereas YAP overexpression reduced these AD hallmarks, and also impacted proteins involved in Aβ generation and tau phosphorylation [20]. Additionally, VGLL4 overexpression in SH-SY5Y cells stably expressing SwAPP695 reduced amyloidogenic processing and protected against synaptic protein loss [18]; however, whether these VGLL4-mediated effects involved Hippo signaling alterations (e.g. reduced TEAD activity through increased VGLL4 competition for YAP) was not assessed. Therefore, the protective effects of VGLL4-overexpression may involve alternative pathways.

Taken together, these studies suggest that, in neurons, Hippo signaling perturbation likely occurs subsequent to initial perturbation in Aβ homeostasis, as the evidence for Hippo signaling regulation of Aβ metabolism is tenuous. However, given that manipulation of YAP levels is able to impact Aβ and tau metabolism in an astrocytic cell line [20], it is possible that astrocytic Hippo signaling alterations may be both a cause and consequence of Aβ toxicity. Moreover, given that YAP-dependent senescence in astrocytes can induce senescence in nearby neurons [21], it is possible that astrocytic alterations could render neurons more vulnerable to injury. Even though Aβ alterations likely precede neuronal Hippo signaling perturbation, Hippo signaling can nevertheless be therapeutically targeted to mitigate further pathological consequences and to slow cognitive decline.

### Upstream mediators of Hippo disruption in AD

The precise mechanisms by which Aβ triggers Hippo pathway disruption in AD are complex and not fully understood. In addition to direct cytoplasmic sequestration of YAP by Aβ [17], evidence suggests that multiple upstream pathways – including cellular stress, metabolic dysregulation, and oxidative damage – may contribute to the dysregulation of various Hippo pathway members in AD. For instance, Aβ could directly induce cellular stress that activates MST1. Oxidative stress, a key feature of AD pathogenesis [136, 168, 169], has been shown to increase MST1 levels, leading to neuronal apoptosis via JNK activation and caspase cleavage [170]. Similarly, oxidative stress can mediate neuronal death through activation of MST1 and FOXO3 transcription factors [126]. Alternatively, Aβ might stimulate upstream kinases like c-Abl, leading to MST1 phosphorylation. Supporting this, c-Abl knockdown in PC12 cells alleviates Aβ-induced apoptosis but not MST1-induced apoptosis, indicating that Aβ may activate MST1 indirectly through upstream kinases such as c-Abl [19]. MST1/2 is also capable of autophosphorylation and so may not require upstream input to initiate Hippo signaling [171]. MST1/2 also serves as a sensor of actin integrity in mammalian cells and can mediate activation of the JNK-p21 pathway in response to actin cytoskeletal disruption [172]. Thus, alterations of the actin cytoskeleton in AD [173] could potentially trigger MST1 activation. In endothelial cells, mtDNA release, which may function as a cellular stress signal in AD [174], has been shown to activate LATS through the cGAS-STING pathway, leading to phosphorylation, nuclear exclusion, and enhanced degradation of YAP [175]. Damaged DNA can also alter YAP activity by enhancing interaction with p73 leading to p73 pro-apoptotic transcriptional activity [46, 48, 49]. Thus, enhanced mtDNA release could contribute to decreased YAP levels in AD. Metabolic alterations are also a crucial feature of the AD brain [176–181]. As both YAP and LATS can respond to changing cellular energy status [141], with glucose starvation leading to AMPK activation of LATS and subsequent phosphorylation and inactivation of YAP [182], reduced glucose levels in AD brains [142, 179, 183], could lead to altered YAP phosphorylation and activity. Levels of O-GlcNAcylation, a post-translational modification that enhances YAP activation by disrupting LATS1/2 phosphorylation and preventing YAP degradation [64, 65], are reduced in AD, possibly due to glucose hypometabolism [184, 185]. This reduction presents another potential mechanism for disruption of Hippo signaling and loss of YAP transcriptional activity in AD.

### Pathological consequences of Hippo activation

Key pathological consequences of Hippo signaling activation relevant to AD pathogenesis include increased mitochondrial dysfunction, oxidative stress, impaired autophagy/mitophagy, and heightened inflammation, which are regulated by various Hippo signaling components.

#### Mitochondrial dysfunction and oxidative stress

Mitochondria are key regulators of cellular homeostasis, contributing to both cellular function and regulation of cell death [186]. Hippo signaling is involved in the maintenance of mitochondrial homeostasis by altering mitochondrial fission, fusion, and biogenesis. In neurons, depletion of upstream Hippo signaling molecule Willin/FRMD6 [6], as occurs with Aβ exposure, leads to mitochondrial dysfunction and morphological alteration [90]. Downstream effector YAP1 influences and responds to mitochondrial signals in several contexts. In HUVE cells, YAP1 activation of TEAD1 increases expression of PGC1α, the master regulator of mitochondrial biogenesis, leading to increased mitochondrial biogenesis, altered mitochondrial morphology, increased glycolysis, and increased angiogenesis [140]. YAP also promotes glycolysis by upregulation of GLUT3, a glucose transporter predominantly found in neurons versus other brain cell types [187]; inhibits gluconeogenesis via PGC1α; and regulates amino acid metabolism by transcriptional stimulation of glutamine-metabolizing enzymes and amino acid transporters (reviewed in [141]). In cardiomyocytes, TEAD1 activation is necessary for expression of nuclear-encoded mitochondrial genes needed for ETC assembly and ATP production; thus, loss of TEAD1 leads to increased mitochondrial ROS, disrupted mitochondrial structure, and impaired energy production [154]. Independent of TEAD transcription factors, YAP also regulates the expression of mitochondrial fusion proteins MFN1/2 [140]. Thus, decreased nuclear YAP levels and reduced YAP-TEAD transcriptional activity in AD could lead to/exacerbate energetic deficits and mitochondrial dysfunction in AD.

Furthermore, oxidative stress is a central feature of AD [136, 168, 169] and is both a cause and a consequence of Hippo pathway activation. In cardiomyocytes, Hippo activation disrupts the interaction between YAP and forkhead box protein O1 (FOXO1), leading to decreased expression of superoxide scavengers catalase and superoxide dismutase 2 (SOD2/MnSOD), which in turn increases oxidative stress, and promotes cell death [188]. Similarly, increased MST1 activation is linked to mitochondrial dysfunction, characterized by excessive mitochondrial fission, impaired mitophagy, and activation of mitochondria-mediated apoptosis, largely through the phosphorylation of mitochondrial fission protein DRP1 [189–191], increasing F-actin assembly through the GSK3β-p53 signaling pathway [191], and altering expression of mitochondrially-encoded respiratory chain subunits by interacting with PGC1α [120]. Activation of MST1 also increases ROS production [120, 192].

#### Impaired autophagy

Autophagy is a critical cellular process that maintains homeostasis by degrading and recycling cellular components [193]. In a *Drosophila* model of polyQ neurodegeneration, the fat/hippo pathway, independent of its well-known functions in cell proliferation and polarity, also plays a role in adult neuronal homeostasis by mediating autophagy [194, 195]. Hippo pathway mutants exhibited autophagic dysfunction, with downstream effector *Yki* being necessary but not sufficient to produce this perturbation [194]. The mechanisms may involve either Hippo-induced activation of mTOR or direct interaction with LC3 proteins [194]. In hepatocellular carcinoma cells, LATS1, but not LATS2, inhibits autophagy by directly binding and stabilizing core autophagy protein Beclin, through increased K27-linked ubiquitination [196]. Notably, this function of LATS1 is independent of its kinase activity, relying instead on a protein domain that is missing from LATS2 [196]. Additionally, a YAP-TEAD inhibitor verteporfin [197, 198], has also been shown to inhibit autophagy [199], further suggesting that Hippo signaling may influence autophagy.

Likewise, Hippo signaling dysregulation in AD can also result in impaired autophagy and mitophagy. In *C. elegans* models of AD, Hippo pathway components affect lysosome morphology and acidification, though the results indicate that decreased, rather than increased, Hippo signaling in this model was associated with autophagic deficits [25]. Specifically, knockdown of LATS1/2 homolog *wts-1* exacerbated abnormal lysosomal morphology in the AD model, while knockdown of YAP and TEAD homologs *yap-1* and *egl-44* attenuated morphological abnormality, reduced autophagosome accumulation, and rescued decreased expression of V-ATPase subunits necessary for lysosome acidification [25]. In other studies using mammalian models, MST1 activation impaired autophagy and promoted apoptosis through the activation of p38-MAPK signaling [111], JNK signaling [200], and FOXO transcription factors [126]. Impairment of autophagy is particularly detrimental in neurons, which are highly dependent on autophagic processes to clear damaged organelles and protein aggregates [201–205].

#### Inflammation

Neuroinflammation including microgliosis and astrogliosis are key contributors to AD pathology [206, 207]. Deletion of YAP in astrocytes has been associated with reactive astrogliosis, microglial activation, and blood-brain barrier dysfunction, through increased JAK-STAT inflammatory signaling [208]. Similarly, in BV2 microglial cells, modulation of YAP levels significantly affects the inflammatory response to Aβ, with YAP overexpression decreasing and YAP knockdown increasing Aβ-induced pro-inflammatory cytokine secretion [14]. VGLL4 is also involved in inflammatory cytokine production in BV2 microglial cells [209]. These results suggest that disruption of YAP-TEAD interaction or decreasing YAP levels can lead to a deleterious pro-inflammatory environment that could exacerbate Aβ toxicity. Through crosstalk between MST1/2 and other important signal transduction molecules such as PKC, FOXO, and Akt, Hippo signaling can also regulate both innate and adaptive immunity [210, 211]. The role of MST1 in microglial activation is also well-documented, with MST1 involved in microglial activation in the brain in response to acute cerebral ischemia-reperfusion injury [212] and chronic unpredictable mild stress (CUMS) [112]. Microglial activation following brain injury is associated with phosphorylation of MST1 leading to increased IκBα and NF-κB signaling, ultimately resulting in neuroinflammation [212]. Inflammatory stimulation of mouse neuronal CATH.a cells increased MST1 levels and subsequent activation of JNK, leading to neuronal apoptosis and mitochondrial dysfunction [200]. Hippocampal overexpression of MST1 in mice also elicited signs of microglial activation [112]. Thus, Hippo signaling alterations such as increased MST1 levels or activation and decreased YAP could contribute to neuroinflammation in the AD brain.

#### Neuronal death and signaling crosstalk

The Hippo signaling pathway is involved in responses to cellular damage through MST1/2 and YAP. YAP inactivation and MST1 hyperactivation can induce cell death via various mechanisms, including caspase activation, JNK signaling, and disruption of Wnt signaling [27, 213–215]. As these signaling pathways have been implicated in AD, crosstalk with dysregulated Hippo pathway components could exacerbate signaling dysfunction in AD, and also suggests that modulating Hippo signaling could influence these other pathways, potentially providing novel therapeutic targets for AD.

Importantly, as MST1 can respond to a wide variety of stress stimuli, this suggests that MST1 serves as a critical node for crosstalk with multiple pro-apoptotic signaling pathways [216]. Activated MST1 can induce cell death through activation of multiple AD-relevant kinases/pathways including LATS1/2, H2B, FOXO3, stress kinase JNK, caspase-3, and p53 [24, 124, 125, 216–223]. In addition MST1 can also activate p38 MAPK [224], though activation of p38 MAPK is not necessary for apoptotic induction by MST1 [225]. Induction of oxidative stress in neuronal HT22 cells leads to increased MST1 levels and subsequent pro-apoptotic changes through activation of JNK and cleaved caspases [170]. An earlier study also demonstrated oxidative-stress induced MST1 activation in primary neurons, which led to apoptosis and neurodegeneration through activation of FOXO transcription factors [126]. Mechanistically, pro-apoptotic activation of MST1 involves its auto-phosphorylation at T183 and subsequent caspase-mediated cleavage, leading to induction of JNK signaling, caspase activation, and mitochondrial-mediated apoptosis [216, 224, 226]. While MST1 cleavage does not appear to be essential for its signal transduction function, MST1 caspase cleavage products themselves have strong pro-apoptotic effects [226], with MST1 and its cleavage products being capable of direct inhibition of the pro-survival kinase Akt1 [216, 227]. Furthermore, JNK can also act upstream of MST1 to enhance pro-apoptotic signaling by phosphorylating MST1 at S82 leading to phosphorylation of FOXO3 at S207 [218]. In *Drosophila* models of neurodegenerative diseases, Hippo signaling activation induces pro-apoptotic effects through enhancement of JNK signaling [110, 118]. MST1 can also exert deleterious effects on hippocampal function through activation of p53 [19]. Direct interaction between MST1 and p53 was demonstrated by IP-MS and coIP studies in HEK293T and neuronal PC12 cells, with the strength of binding enhanced by MST1 kinase activity [19]. Enhanced activation of p53 by MST1 contributes to neuronal death in vitro and cognitive decline in vivo [19].

Perhaps best-studied of these signaling pathway interactions is the crosstalk between Hippo and Wnt signaling, which can occur with both MST and YAP/TAZ. In Wnt-OFF cells, cytoplasmic YAP/TAZ function as integral components of the β-catenin destruction complex, resulting in the exclusion of both YAP/TAZ and β-catenin from the nucleus and the shutting off of their respective transcription programs [228]. Cells in the Wnt-OFF state may therefore phenocopy Hippo-ON cells, highlighting the potential for targeting dysregulated Wnt signaling in AD through modulating Hippo signaling, particularly as decreased Wnt signaling is associated with age-related cognitive decline and AD [214]. Likewise, further studies have demonstrated that Hippo signaling antagonizes Wnt signaling by reducing nuclear β-catenin levels [229, 230]. Conversely, activation of Wnt signaling increases YAP nuclear localization and activity while increasing TAZ stability [74, 228, 231]. As a caveat, the details of this crosstalk are still poorly understood, as studies have shown differing effects of YAP on β-catenin nuclear translocation and activation (reviewed in [44]). Crosstalk between Wnt and Hippo is further underlined by studies demonstrating that inhibition of core Hippo kinase MST1/2 can also affect Wnt signaling components Wnt-3a, GSK3β, and β-catenin [15].

### Hippo pathway inhibition for the treatment of AD

Therapeutic strategies for neurodegenerative disorders often focus on blocking neuronal cell death due to the limited regenerative capacity of neurons [27]. As discussed above, neurodegeneration in AD patients and rodent models is associated with activation of Hippo signaling; thus, Hippo pathway modulation has emerged as a promising target for therapeutic intervention. Recent studies demonstrate that general inhibition of Hippo signaling can reverse cognitive decline and pathological changes in AD models. For example, treatment with an *Artemisia annua* extract reverses signs of Hippo signaling activation and improves cognitive decline and pathological changes in 12-month-old 3xTg mice [24], suggesting the potential benefits of Hippo signaling inhibition. More specific modulation of the Hippo pathway could be achieved through a variety of approaches including inhibition of MST1/2 kinase, alteration of YAP localization, and disruption of YAP-TEAD interaction [27]. Given the cascade of pathological consequences linked to Hippo activation in AD – such as oxidative stress, mitochondrial dysfunction, impaired autophagy, and inflammation – inhibiting this pathway could be a viable strategy to halt disease progression. However, potential side effects, given the tumor suppressive role of Hippo signaling in multiple tissues, remain an area of concern for such studies. The following sections will explore current findings on the effects of Hippo pathway inhibition in AD, examining its potential benefits and associated challenges.

#### Targeting MST1

A majority of current studies have focused on inhibiting MST1, which is an attractive target given its central role in responding to stress signals and regulating neuronal survival and apoptosis through a variety of signaling pathways. Indeed, in vivo studies involving AAV-mediated MST1 overexpression in the hippocampus of WT mice, indicate that MST1 overexpression alone is sufficient to produce deleterious neurodegenerative phenotypes, including cognitive impairments, synaptic dysfunction, disruption of hippocampal neural networks, neuronal apoptosis, neuronal loss, and synaptic loss [19, 112, 232]. Mechanistically, MST1 overexpression increases phosphorylation of both YAP and FOXO3a as well as expression of pro-apoptotic bcl-2 and bax [232]. Furthermore, increased MST1 activity is found in neurodegenerative diseases including HD [11], PD [233], and ALS [111]. By contrast, while MST1 dysregulation has been reported in AD patient serum [234], increased MST1 phosphorylation/activation has yet to be reported in AD patient brains. Despite this, multiple studies indicate an important role for MST1-mediated neuronal dysfunction leading to cognitive deficits and neuronal loss in AD models. For instance, expression of MST1/2 homolog *hpo* in a *Drosophila* eye model of AD enhances the neurodegenerative phenotype [118]. Likewise, in the 5xFAD mouse model, MST1 overexpression leads to earlier onset of cognitive deficits and AD pathology [19, 120].

Various in vivo studies have demonstrated protective effects of modulating Hippo signaling through MST1 in neurodegenerative disorders. MST1 inhibition or depletion protects against neurodegeneration in multiple rodent models of neurodegenerative conditions, including ALS, where MST1 knockout reduced neurodegeneration [111]; CUMS-induced depression, where MST1 knockdown rescued increased p38 activation in response to chronic stress and protected against stress-induced depression-like behaviors, impairment of synaptic plasticity [112], and altered neural oscillation patterns [235]; and traumatic brain injury (TBI), where administration of CGP3466B protected against neuronal apoptosis by inhibiting MST1 via PCMT1 [236]. These studies demonstrate that decreasing MST1 levels or activity can protect against neurodegeneration. This is also the case in AD models. In vitro experiments in Aβ-treated PC12 [19] and SH-SY5Y [120] neuronal cells demonstrate that MST1 knockdown reverses Aβ-induced increases in apoptotic markers. Additionally, in a *Drosophila* eye model of AD, blockade of Hippo signaling by knockdown of *hpo* ameliorates Aβ-mediated cell death and JNK activation [118], suggesting a synergistic relationship between these two pathways in an Aβ-rich environment. Interestingly, the authors found that altering *hpo* levels did not affect Aβ accumulation/levels [118]. Thus, this study demonstrated that while Hippo dysregulation may not initiate Aβ accumulation in AD, modulation of Hippo signaling could still mitigate Aβ-induced cell death.

The therapeutic potential of MST1 inhibition in AD is further supported by the success of the MST1/2 inhibitor XMU-MP-1 in various experimental models. The neuroprotective effects of XMU-MP-1 may involve modulation of MST1/2-mediated pro-apoptotic signaling cascades through both YAP-dependent and independent mechanisms. MST1 knockdown or pharmacological inhibition by XMU-MP-1 ameliorates cognitive deficits and pathological changes in 5xFAD mice through YAP-independent mechanisms including decreased pro-apoptotic p53 activation [19], increased PI3K-Akt activation, and increased mitochondrial biogenesis [120]. As MST1 kinase activity is required for its cleavage by caspases [224] and as caspase-cleaved MST1 can translocate to the nucleus to amplify apoptotic responses [237], XMU-MP-1 could also dampen the apoptotic response by decreasing caspase-mediated cleavage of MST1 through inhibition of its kinase activity. MST1 also activates both p38 MAPK and JNK to amplify apoptotic signaling [224], suggesting that MST1 inhibition may also protect against neuronal death through modulation of these signaling pathways.

In APP/PS1 mice, XMU-MP-1 administration protects against cognitive deficits and reverses signs of astrocytic senescence through a mechanism requiring YAP [21], demonstrating that MST1 inhibition is also beneficial in non-neuronal brain cell types. Supporting this, in vitro studies demonstrate that XMU-MP-1 can also impact microglial activation; thereby influencing the pro-inflammatory environment of the AD brain. Specifically, pharmacological inhibition of MST1/2 reduces, while disruption of YAP-TEAD interaction by verteporfin exacerbates Aβ-induced proinflammatory cytokine secretion in BV2 cells and primary microglia [14]. The detrimental effect of verteporfin is intriguing given that verteporfin has also been investigated as a possible AD drug lead given its alternative function as a γ-secretase inhibitor [238].

Thus far we have mainly focused on Aβ; however, XMU-MP-1 may also be effective against tau pathology as demonstrated in an STZ-induced rat model of sporadic AD, where XMU-MP-1 administration protected against tau hyperphosphorylation, Aβ deposition, oxidative stress, acetylcholinesterase hyperactivity, neuroinflammation, loss of synaptic proteins, increased apoptosis, and neuronal loss and morphological changes [15]. XMU-MP-1 also reversed changes associated with Hippo activation including increased phosphorylation of MST1/2, LATS1/2, and YAP, and decreased nuclear translocation of YAP [15]. Notably, XMU-MP-1 rescued cognitive decline to an extent comparable to that achieved by approved AD drug donepezil [15]. Intriguingly, XMU-MP-1 also restored Wnt signaling in the STZ-induced AD rat model [15], suggesting that its therapeutic effects may involve reactivation of both β-catenin and YAP transcriptional programs.

In addition to impacts on aberrant signal transduction, XMU-MP-1 can also modulate protein-protein interactions. For example, XMU-MP-1 can disrupt the interaction between LATS1/2 and KIBRA, demonstrating a YAP-independent mechanism through which MST1 inhibition can impact hippocampal neuronal networks and AMPAR regulation leading to improved spatial and object memory in male ArcAβ mice [16]. Mechanistically, XMU-MP-1 inhibition of MST1/2 reduces LATS1/2 phosphorylation and disrupts its interaction with KIBRA, thus increasing KIBRA availability for scaffolding AMPAR complexes and preserving synaptic function and cognition [16]. Intriguingly, unlike previous studies in AD rodent models that demonstrated an effect of XMU-MP-1 on YAP nuclear localization [15] and transcriptional activity [21], Stepan et al. [16]. found that XMU-MP-1 did not affect YAP-TEAD downstream targets. While the discrepancy could have resulted from the use of different AD models, the more likely explanation lies in their treatment paradigms. The studies demonstrating an effect on YAP administered XMU-MP-1 at comparable dosages over a two-week period [15, 21], whereas Stepan et al. [16]. evaluated YAP-TEAD target gene expression 45 min after administration of a single higher dosage, suggesting that *prolonged* but not acute XMU-MP-1 treatment induces changes in YAP transcriptional activity. Though further studies are needed to characterize the details of XMU-MP-1 modulation of downstream transcriptional activity, the study by Stepan et al. [16]. nevertheless provides encouraging evidence that AMPAR signaling and cognitive decline can be targeted through modulating Hippo signaling, and that this could potentially be performed in a manner that does not affect downstream Hippo signaling, which could help to prevent off-target effects associated with inhibition of a known tumor suppressor pathway. XMU-MP-1 could also modulate interactions between other Hippo pathway members. For example, a recent study in fibroblasts demonstrated that the interaction of upstream Hippo pathway regulator Willin/FRMD6 with MST1/2, which leads to YAP inactivation and induction of cellular senescence, can also be abrogated by XMU-MP-1 [87].

One caveat for the usage of XMU-MP-1 is the potential for off-target effects, as a kinobead assay in SH-SY5Y neuroblastoma cells demonstrated that XMU-MP-1 can potentially inhibit other kinases including AURKA/B, TAOK2, MAPK7, and JAK1 [16]. Though it is possible that the neuroprotective effects of this small molecule inhibitor may also be mediated through effects on these other kinases, further studies are needed to determine whether and how XMU-MP-1 administration may impact these off-target kinases and the potential consequences of their inhibition.

#### The benefits and challenges of targeting a tumor suppressor pathway

Given that Hippo signaling is a known tumor suppressor pathway, its emerging role in AD raises two important considerations. First, there exists the possibility for drug repurposing of anticancer drugs to modulate the pathway for AD therapeutics. For example, studies in AD models have used anti-cancer drug Gleevec (ST1571) to inhibit tyrosine kinase c-Abl, an upstream regulator of Hippo signaling. c-Abl can contribute to pro-apoptotic signaling through phosphorylation of MST1 at Y433, leading to enhanced interaction with FOXO3 [239], and YAP1 at Y357, leading to decreased TEAD activity [50] and enhanced interaction with p73 [51]. Levels of c-Abl are significantly increased in AD mouse models and following Aβ treatment of primary neurons [240–242]. Promisingly, inhibiting c-Abl using Gleevec (ST1571) decreases Aβ secretion and enhances activity of Aβ-degrading enzyme neprilysin in vitro [243], and shifts APP processing towards nonamyloidogenic cleavage in vivo in 3xTg mice [244]. Other potential pharmacological inhibitors of Hippo signaling that could be explored as therapeutic avenues for AD are listed in Table 3.

**Table 3:** Hippo pathway pharmacological inhibitors

Inhibitor	Mechanism of action	Use in Alzheimer’s disease?	Potential translational applications
XMU-MP-1	MST1/2 inhibitor	Multiple studies	Promotion of intestinal repair and rescue of bone marrow failure following high dose irradiation in cancer [245]. Treatment of osteoporosis [246].
VT02956	LATS inhibitor	No reports	Treatment of estrogen receptor-positive breast cancer [247].
TRULI	LATS inhibitor	No reports	No reports
GA-017	LATS inhibitor	No reports	No reports
ION537	YAP antisense oligonucleotide	No reports	Phase I clinical trial for treatment of solid tumors [248].
CA3	YAP expression inhibitor	No reports	Treatment of hepatocellular carcinoma, in combination with sorafenib [249]. Potential applications in treatment of mesothelioma [250].
Verteporfin	YAP-TEAD interaction inhibitor	Poor candidate due to cytotoxicity and bioavailability; inhibits γ-secretase [238]	Used during photodynamic therapy for pathological myopia, ocular histoplasmosis and choroidal neovascularisation in macular degeneration [251]. Potential applications in treatment of mesothelioma [250].
VGLL4 peptide mimic	YAP-TEAD interaction inhibitor	No reports	Treatment of gastric cancer [252].
Peptide 17	YAP-TEAD inhibitor	No reports	No reports
IAG933	YAP/TAZ-TEAD inhibitor	No reports	Potential applications in Hippo-pathway driven mesothelioma, as well as Hippo altered cancer models like lung, pancreatic, and colorectal cancer [253].
BPI-460372	TEAD inhibitor	No reports	Potential applications for treatment of solid tumors with Hippo pathway alterations, such as mesothelioma, meningioma, soft tissue sarcoma, and non-small cell lung cancer [254].
MYF-01-37	TEAD inhibitor	No reports	No reports
Flufenamic acid and derivatives	TEAD inhibitor	No specific reports. Part of a class of NSAIDs that have been explored for AD and other neurodegenerative disorders [255].	Potential applications in breast cancer treatment [256].
MGH-CP1	TEAD2/4 inhibitor	No reports	No reports
VT3989	TEAD inhibitor	No reports	In clinical trial for treatment of advanced solid tumors in malignant mesothelioma and other tumors with NF2 mutations [257].
IK-930	TEAD inhibitor	No reports	No reports

Second, the canonical role of Hippo signaling in restricting cell proliferation and preventing oncogenic transformation [29] demonstrates a potential for off-target effects of inhibiting this pathway for AD treatment. Inhibition of Hippo kinases, while beneficial for boosting YAP activity and neuronal survival in AD models, may carry a risk of promoting tumorigenic processes, particularly as Hippo signaling crosstalks with other tumor suppressor pathways including p53 [258], PI3K-Akt-mTOR [227, 259, 260], and Wnt [228–230, 261]. While complete and untargeted inhibition of Hippo signaling is unlikely to be safe for long-term treatment, strategies to mitigate the risk of tumorigenesis could include the use of cell-type specific therapeutics. For example, as cancers of the nervous system are typically found in glia, with neuronal brain tumors being exceedingly uncommon and usually low-grade and minimally aggressive [262], targeting Hippo inhibitors to neurons decreases the risk of oncogenesis. Targeting neurons is also sensible as neuronal Hippo activation plays a significant role in AD pathogenesis. Furthermore, appropriate timing of the treatment course could help to avoid increased risk from prolonged suppression of Hippo signaling, implying the need for developing/monitoring both oncogenic and AD-progression related biomarkers. Differences in results from administration of XMU-MP-1 in AD mice with regards to YAP activation suggest that while acute treatment with XMU-MP-1 appears sufficient to ameliorate cognitive deficits without affecting YAP transcriptional output [16], longer treatment regimes produce alterations in YAP localization or activity [15, 19], which carries increased possibility of off-target effects. In this vein, the cancer field has developed reversible small molecule inhibitors to selectively modulate proteins such as p53 to partially inhibit its action without fully inactivating its tumor suppressor role. For example, pifithrin-α (PFT-α) blocks p53 transactivation of select target genes without affecting basal levels by acting on p53 PTMs [263, 264]. The development of similar inhibitors for suppressing Hippo activation could also decrease oncogenic risk. Lastly, developing inhibitors targeting PTMs that act as *modulators* of Hippo signaling strength could also be used as a strategy to preserve tumor suppressive function. For example, O-GlcNacylation appears to be isoform specific with regards to Hippo kinases; therefore, targeting O-GlcNacylation may be a method to diminish, but not completely deplete, Hippo signaling. Furthermore, Tanaka et al. [17]. reported that AAV-mediated administration of neuronal-specific YAP isoform YAP-deltaC did not result in tumors in mice at 6 months follow-up [17], suggesting that targeting neuron-specific isoforms could also be used to decrease chances of tumorigenesis.

## Conclusions

Neuronal death during AD pathogenesis involves dysregulation of the Hippo signaling pathway, with activation of Hippo signaling associated with neuronal loss during early stages of the disease. Aβ has been shown to induce phosphorylation of MST1/2, leading to pro-apoptotic changes including JNK activation, caspase cleavage, p53 activation, and phosphorylation and cytoplasmic sequestration of YAP leading to reduced YAP-TEAD interaction. Consequently, inhibition of the Hippo signaling pathway, particularly through targeting MST1/2, presents a promising therapeutic avenue for AD. Evidence from various preclinical models indicates that modulation of this pathway can alleviate key pathological features of AD, including neuroinflammation, oxidative stress, synaptic dysfunction, and neuronal apoptosis. Pharmacological inhibitors like XMU-MP-1 have demonstrated significant neuroprotective effects in vivo. Furthermore, the potential to repurpose small molecules from cancer and tissue regeneration research for AD treatment broadens the therapeutic possibilities. Indeed, despite a growing list of small molecules targeted towards various Hippo pathway members (Table 3), studies in the AD field thus far have focused on MST1 inhibitor XMU-MP-1.

Although studies have now begun to demonstrate strong evidence for Hippo signaling dysregulation in AD, several key questions remain unanswered. First, it will be essential to determine when, where, and how AD pathology specifically activates or suppresses this pathway to enable effective therapeutic targeting. Given that studies demonstrate the potential existence of a hypo-Hippo phase in late-stage AD, though the evidence in AD patient tissues is based mainly on transcriptomic data, the successful implementation of Hippo inactivation as a therapeutic strategy will hinge on the elucidation of the spatio-temporal characteristics of this putative phase. Specifically, studies will need to delineate if and when this phase occurs during AD pathogenesis. In this vein, MST presents an attractive initial therapeutic target, as studies in mammalian models have consistently reported its activation in AD. For Hippo components with more varied alterations, a biomarker for the switch from hyper to hypo-Hippo phases will need to be identified to appropriately time treatments. Tanaka et al. [17]. demonstrate that levels of HMGB1, which is released by necrotic cells, are significantly elevated in the CSF of MCI, but to a lesser extent in AD patients, mirroring the increases in YAP-dependent neuronal necrosis in the early stage of the disease. Furthermore, as the expected outcome of decreased KIBRA levels is hypo-Hippo signaling and as decreasing KIBRA levels are associated with increasing tau pathology [121], changes in tau could potentially be used along with HMGB1 levels to gauge the appropriate timing of Hippo signaling inhibition as a treatment strategy. Second, it will be vital to explore the roles of TAZ in different brain cell types and whether these act in tandem or in opposition to YAP. Third, while YAP-TEAD transcriptional activation forms the canonical output of Hippo signaling, YAP can also bind to non-TEAD transcription factors and induce alternate transcription programs [44, 265, 266]; thus, characterizing transcriptional activation in response to AD-related alterations in YAP levels and YAP subcellular localization could help to further elucidate the mechanisms of YAP-driven cell death or survival. Of particular interest is p73, which has multiple isoforms – produced through alternative promoter usage and splicing – with both pro-apoptotic and anti-apoptotic effects [267, 268]. Defining whether and how these isoforms affect YAP-p73 outputs, and whether this is disrupted in AD, could help elucidate the role of YAP-p73 interaction in neurodegeneration.

## Data Availability

Not applicable.

## Abbreviations

MST1/2: Mammalian Ste20-like kinases 1 and 2
LATS1/2: Large tumor suppressor kinases 1 and 2
YAP: Yes-associated protein
TAZ: Transcriptional coactivator with PDZ-binding motif
SAV1: Protein Salvador homolog 1
MOB1A/B: Mps one binder kinase activator-like 1 A/B
KIBRA: WW domain-containing protein 1
TEAD1-4: TEA domain family member 1-4
ECM: Extracellular matrix
SNPs: Single nucleotide polymorphisms
AD: Alzheimer’s disease
ALS: Amyotrophic lateral sclerosis
HD: Huntington’s disease
Crb: Crumbs
APP: Amyloid precursor protein
TRIAD: Transcriptional repression-induced atypical cell death
Plk1: Polo-like kinase 1
PD: Parkinson’s disease
CUMS: Chronic unpredictable mild stress
TBI: Traumatic brain injury
MCI: Mild cognitive impairment
WT: Wild type
